# Neutrophil activation and NETosis are the predominant drivers of airway inflammation in an OVA/CFA/LPS induced murine model

**DOI:** 10.1186/s12931-022-02209-0

**Published:** 2022-10-21

**Authors:** Mengling Xia, Fei Xu, Hangqi Ni, Qing Wang, Ruhui Zhang, Yafang Lou, Jianying Zhou

**Affiliations:** 1 Department of Respiratory Disease, Thoracic Disease Center, The First Affiliated Hospital, College of Medicine, Zhejiang University, No. 79, Qingchun Road, 310003 Hangzhou, China; 2 Department of Respiratory Medicine, Hangzhou Hospital of Traditional Chinese Medicine, No. 453, Tiyuchang Road, 310013 Hangzhou, China

**Keywords:** Neutrophilic asthma, Neutrophil, Neutrophil extracellular traps, Airway inflammation

## Abstract

**Background:**

Asthma is one of the most common chronic diseases that affects more than 300 million people worldwide. Though most asthma can be well controlled, individuals with severe asthma experience recurrent exacerbations and impose a substantial economic burden on healthcare system. Neutrophil inflammation often occurs in patients with severe asthma who have poor response to glucocorticoids, increasing the difficulty of clinical treatment.

**Methods:**

We established several neutrophil-dominated allergic asthma mouse models, and analyzed the airway hyperresponsiveness, airway inflammation and lung pathological changes. Neutrophil extracellular traps (NETs) formation was analyzed using confocal microscopy and western blot.

**Results:**

We found that the ovalbumin (OVA)/complete Freund’s adjuvant (CFA)/low-dose lipopolysaccharide (LPS)-induced mouse model best recapitulated the complex alterations in the airways of human severe asthmatic patients. We also observed OVA/CFA/LPS-exposed mice produced large quantities of neutrophil extracellular traps (NETs) in lung tissue and bone marrow neutrophils. Furthermore, we found that reducing the production of NETs or increasing the degradation of NETs can reduce airway inflammation and airway hyperresponsiveness.

**Conclusion:**

Our findings identify a novel mouse model of neutrophilic asthma. We have also identified NETs play a significant role in neutrophilic asthma models and contribute to neutrophilic asthma pathogenesis. NETs may serve as a promising therapeutic target for neutrophilic asthma.

**Supplementary Information:**

The online version contains supplementary material available at 10.1186/s12931-022-02209-0.

## Introduction

Asthma is a heterogeneous chronic respiratory disease, characterized by airway hyperresponsiveness (AHR) and airway inflammation [1]. There are four main inflammatory phenotypes in asthma based on the proportion of granulocytes in induced sputum: neutrophilic asthma, eosinophilic asthma, paucigranulocytic asthma, and mixed granulocytic asthma [2]. Neutrophilic asthma is one of the main types of severe asthma [3], which exhibits worse lung function, more severe airway inflammation, and worse treatment response. Therefore, it is crucial to investigation the pathogenesis of neutrophilic asthma.

Experimental animal models are essential to advance asthma pathophysiological research, and mice are the main subjects. There are classical animal models of eosinophilic asthma, such as ovalbumin (OVA)/ aluminum (Alum)-sensitized and OVA-challenged model of asthma, house dust mite (HDM) extract-induced model of asthma and so on [4–7]. However, to the best of our knowledge, there is no animal model of neutrophilic asthma to be universally accepted. Currently, there are some animal studies of neutrophilic asthma, for example, OVA/complete Freund’s adjuvant (CFA)-sensitized and OVA-challenged model of asthma, OVA/ lipopolysaccharide (LPS)-sensitized and OVA-challenged model of asthma [8–10]. While these models recapitulate some of the features of human neutrophilic asthma, each of these models had certain limitations.

Neutrophils are the main effector cells of inflammation and tissue infection [11]. The formation of neutrophil extracellular traps (NETs) is a function of neutrophils [12]. Neutrophils increase when inflammation occurs, and can undergo a process of NETosis, releasing NETs outside the cells [13]. However, whether NETs are generated and function in neutrophilic asthma have not been completely elucidated.

Our study demonstrated that the OVA/CFA/LPS-induced murine model is suitable for study as a model of neutrophilic asthma. This kind of model has massive neutrophilic inflammation and severe airway hyperresponsiveness. In addition, we also found that a large number of NETs were produced and correlated with the severity of airway inflammation. Therefore, it is reasonable to hypothesize that NETs play an important functional role in neutrophilic asthma.

## Materials and methods

### Animals

BALB/c mice (female, 6-8 weeks) were purchased from Hangzhou Medical College (Hangzhou, China). We maintained all the mice in specific pathogen-free (SPF) conditions with a 12 light/12 dark cycle in the Experimental Animal Center, the First Affiliated Hospital, School of Medicine, Zhejiang University.

### Mouse allergen sensitization and challenge

Mouse models were induced using modified protocols as previously reported [7–9]. Mice were randomly grouped and sensitized on day 1 and day 8. For the OVA/Alum group of asthma, 25 µg OVA (A5503, Sigma Aldrich, St. Louis, MO, USA) dissolved in 100 µl 0.9% saline was mixed 1:1 with Imject Alum (Pierce, Rockford, IL, USA) by intraperitoneal (IP) injection. For the OVA/CFA group of asthma, 25 µg OVA dissolved in 100 µl 0.9% saline was mixed 1:1 with CFA (F5881, Sigma Aldrich, St. Louis, MO, USA) by IP. injection. For the OVA/LPS group of asthma, mice were lightly anesthetized with isoflurane, and intratracheally injected using a combination of 25 µg OVA with 0.1 or 10 µg LPS (L2630, Sigma Aldrich, St. Louis, MO, USA) in a total volume of 40 µl, with 0.9% saline as the diluent. For the OVA/CFA/0.1 LPS group, the model of sensitization is a combination of the OVA/CFA group and OVA/LPS group. After sensitization, the above-mentioned groups were challenged with aerosolized 1% OVA for 30 min on days 15-17. For the OVA/CFA/0.1 LPS + DNase I group or the OVA/CFA/0.1 LPS + CI-amidine group, on the basis of the OVA/CFA/0.1 LPS group, intravenous injection of DNase I (5 mg per kg body weight) or intraperitoneal injection of Cl-amidine (10 mg per kg body weight) was performed 1 h before each challenge. Mice sensitized and challenged with 0.9% saline were used as controls.

### Airway hyperresponsiveness measurement

Airway responsiveness was assessed 24 h after the last OVA challenge as previously described [14]. Briefly, mice were placed in a plethysmograph chamber (EMKA Technologies, Paris, France) for at least 10 min to adaption. Baseline pulmonary parameters were first determined, and then mice were sequentially challenged with aerosolized PBS and methacholine (Mch, A2251, Sigma Aldrich, St. Louis, MO, USA) at increased concentrations (3.125, 6.25, 12.5, 25 and 50 mg/ml in PBS). Enhanced Pause (Penh), an indirect estimate of airway resistance, was used to measure airway resistance to methacholine. After each nebulization, record the Penh value for 3 min and take the average of three consecutive values.

### Bronchoalveolar lavage

Mice were euthanized 48 h after the last OVA challenge, serum, bronchoalveolar lavage fluid (BALF), bone marrow and lung tissues were collected for further experiments. As described previously [14], BALF was collected on whole lungs by infusing 1 ml of PBS via a tracheal cannula. Then, the BALF was centrifuged, and the supernatants were stored at-80 °C until analysis, the cell pellet was resuspended in PBS for differential cell counting or flow cytometry analysis.

### Cytokine analysis

Expression of interleukin (IL)-4 (431,104), IL-17 A (432,504), IL-6 (431,304), and IL-1β (432,604) in BALF were quantitated by enzyme-linked immunosorbent assay (ELISA) kit (Biolegend, San Diego, CA, USA) according to the manufacturers’ instructions.

### Lung histopathology staining

After the BALF was collected, the right main bronchus was ligated, and the left lung was perfused and fixed with 4% paraformaldehyde for 24 h, followed by paraffin embedded, sectioned, stained with hematoxylin and eosin (H&E) or paraffin acid-Schiff (PAS) staining. Sections were performed in a blinded fashion. The degree of inflammation on H&E-stained lung sections was scored as described previously [15]: 0, normal; 1, few inflammatory cells; 2, a ring of inflammatory cells 1 cell layer deep; 3, a ring of inflammatory cells 2 to 4 cells deep; 4, a ring of inflammatory cells greater than 4 cells deep. Mucus-containing goblet cells on PAS-stained lung sections was also scored previously described [16]: 0, no PAS-positive cells; 1, less than 25%; 2, 25 to 50% PAS-positive cells; 3, 50 to 80% PAS-positive cells; 4, greater than 80% PAS-positive cells.

### Flow cytometry analysis

Flow cytometry of leukocytes in BALF, lung tissue, peripheral blood and bone marrow. The BALF were collected according to the above procedure. Lung single-cell suspensions were obtained as previously described [17]. The peripheral blood was collected from mice by removed their eyeball and placed in tubes containing EDTA. Red blood cells (RBCs) were lysed with RBC lysis buffer (Biolegend, 420,301, San Diego, CA, USA). The bone marrow cells were harvested from the femora and tibiae by flushing with PBS, then cells filtered through 40 μm strainers (352,340, BD Biosciences, San Diego, CA, USA) to obtain single cell suspension. After a washing step, all single-cell suspensions were incubated with Fixable Viability Stain 780 (FVS780, 565,388, BD Biosciences, San Diego, CA, USA, 1:1000) according to manufacturer’s instructions to gate out dead cells. Subsequently, samples were stained with antibodies against CD45-FITC (157,214, Biolegend, San Diego, CA, USA, 1:100), CD11b-PE (101,207, Biolegend, San Diego, CA, USA, 1:100) and Ly6G-PE/Cy7 (560,601, BD Biosciences, San Diego, CA, USA, 1:100) for 30 min on ice, then cells were analyzed on a Cytoflex LX flow cytometer (Beckman Coulter, CA, USA). All above antibodies were diluted in PBS. Analyses were performed using FlowJo software v10.6.2. Neutrophils were identified as CD45(+)CD11b(+)Ly6G(+).

### Immunostaining and confocal microscopy

Bone marrow cells from mice were harvested as describe above. Subsequently, neutrophils from bone marrow were isolated by density gradients according to the mouse neutrophil isolation kit instructions (TBD2013NM, TBD Science, China). The neutrophils (2 × 10^5^ cells per well in serum free RPMI 1640) were plated on poly-lysine-coated round coverslips and placed in 24-well culture plates. Then, the cells were stimulated with 100 nM Phorbol 12-myristate 13-acetate (PMA, P3681, Sigma Aldrich, St. Louis, MO, USA) or RPMI 1640 (Ctrl) for 4 h. Subsequently, cells were fixed in 4% paraformaldehyde (PFA) for 30 min and permeabilized by incubation in 0.5% Triton X-100 at room temperature (RT) for 1 min. After that some samples were stained with propidium iodide (PI, 5 µg/ml, P4170, Sigma Aldrich, St. Louis, MO, USA) for 30 min, and washed in PBS before confocal microscopic observation. To quantify NET formation, NETosis was defined as neutrophils with flattened nuclei, decondensed chromatin and expulsion of extracellular neutrophils under fluorescence microscopy. Two independent researchers analyzed 200 neutrophils in each sample [18]. The other samples were blocked by blocking solution (phosphate-buffered saline (PBS) containing 1% bovine serum albumin (BSA) and 0.1% Tween-20) for 1 h at RT, samples were stained with rabbit anti-citrullinated histone 3 (CitH3) (ab5103, Abcam, 1:500) and mouse anti- Myeloperoxidase (MPO) (AF3667, R&D, 1:50) overnight at 4 °C. The next day, samples were washed in PBST and incubated with secondary antibodies CoraLite 594-conjugated Goat Anti-Rabbit IgG (H + L) (Proteintech, SA00013-4, 1:400) and CoraLite 488-conjugated Affinipure Goat Anti-Mouse IgG (H + L) (Proteintech, SA00013-1, 1:400). All above antibodies were diluted in blocking solution. The nuclei were stained with 4,6-diamidino-2-phenylindole (DAPI, 422,801, Biolegend 1:10000). The NETs were identified by the colocalization of antibodies (MPO, CitH3 and DAPI) and quantified using Fiji software v2.1.0. To determine the colocalization, we used a ratio by normalizing the percentage CitH3 signal to the percentage MPO signal, analysis of the CitH3/MPO percentage values from four regions was performed [19].

### Western blot

Protein lysates of lung tissue were prepared. Then, the proteins were separated by 10-12% sodium dodecyl sulfatepolyacrylamide gel electrophoresis (SDS-PAGE), transferred onto polyvinylidene fluoride (PVDF) membranes. Membranes were blocked in 5% skim milk for 1 h RT, follow by incubated with primary antibodies overnight at 4 ℃. The following day, membranes were washed and incubated with HRP-conjugated secondary antibodies for 1 h and proteins were visualized using the ECL reagent. Rabbit anti-CitH3 (ab5103, 1:1000) was purchased from Abcam; Mouse anti-MPO (AF3667, 1:400) was purchased from R&D; beta (β)-Tubulin (66,240–1-Ig, 1:5000) was purchased from Proteintech Technology. All primary antibodies were diluted in primary antibody dilution buffer (Beyotime Biotechnology, China), and secondary antibodies were diluted in 5% skim milk.

### Statistical analysis

All statistics and graphs were performed using GraphPad Prism software v8.4.0 (GraphPad Software Inc., San Diego, CA, USA). In this study, a one-way ANOVA with Tukey posttests was used for multiple comparisons. Data are presented as means ± standard errors of measurement (SEM). Significant differences are shown as *P < 0.05, **P < 0.01, and ***P < 0.001.

## Results

### AHR and pathological changes induced by different allergens combined with OVA

**Fig. 1:**
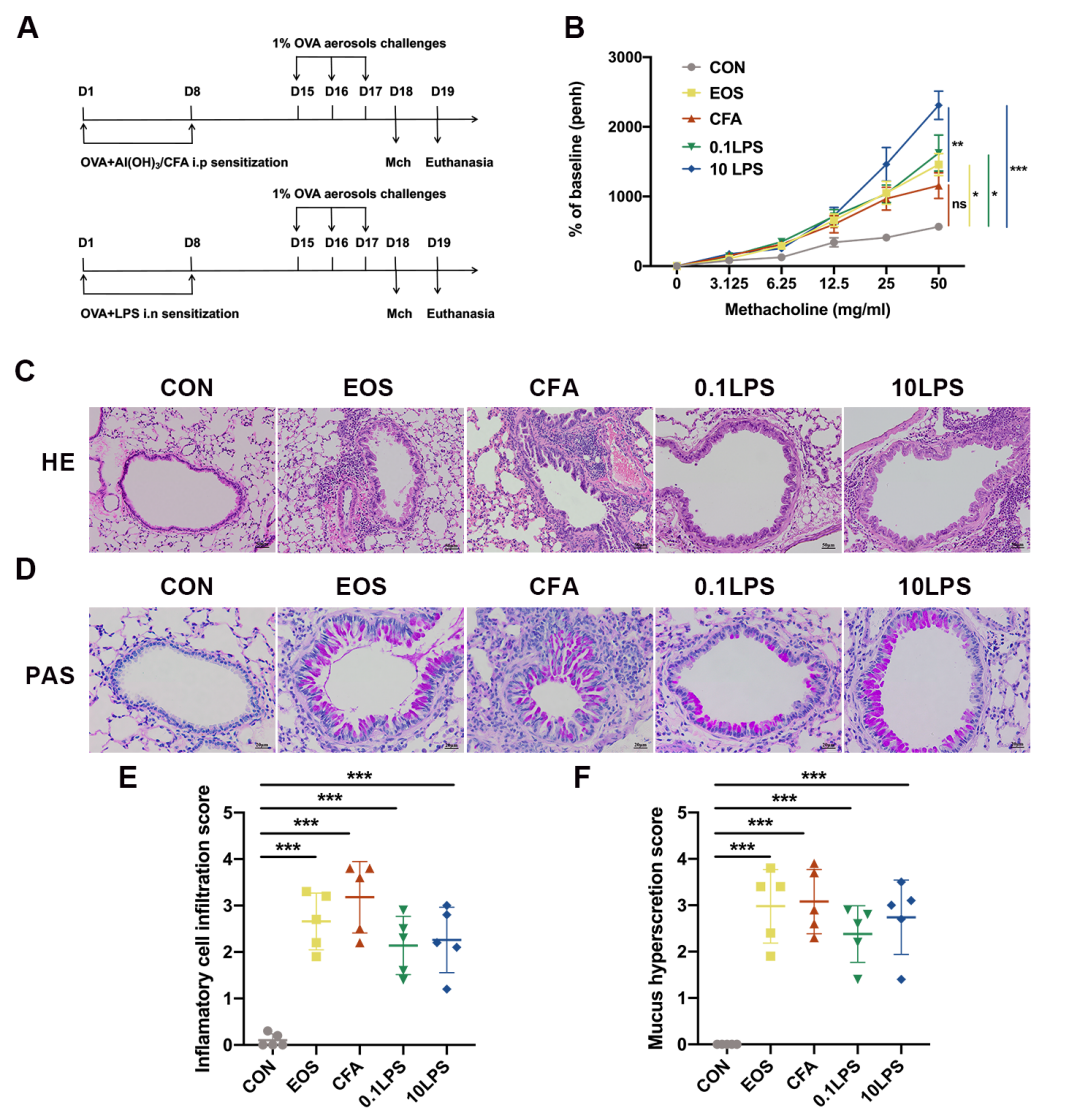
Different allergens combined with OVA-induced airway hyperresponsiveness (AHR) and pathological changes. (A) Schematic representation of experimental procedure. (B) The AHR was determined by calculating enhanced pause (Penh) values 24 h after the last challenge. (C) Hematoxylin and eosin (H&E) staining of lung tissue. Scale bar = 50 μm. (D) Paraffin acid-Schiff (PAS) staining of lung tissue. Scale bar = 20 μm. (E) Quantification of inflammation infiltration score of the H&E staining. (F) Quantification of mucus-producing goblet cells of the PAS staining. Data were shown as mean ± SEM; n = 5. Significance between groups was calculated using one-way ANOVA with Tukey’s post hoc method. *p < 0.05, **p < 0.01 and ***p < 0.001

To establish a suitable model to recapitulate as many clinical features as possible of human severe neutrophilic asthma, we constructed three different type of neutrophilic asthma model in BALB/c mice using CFA/OVA or LPS/OVA. In parallel, we established an eosinophilic asthma model as a control using Alum/OVA (Fig. 1A). Whole-body plethysmography was used to assess the AHR. The 10 µg LPS/OVA (10LPS) group exhibited a drastic increase in Penh upon exposure to increasing concentrations of aerosolized methacholine, as compared to the saline-treated (control) control group and CFA/OVA (CFA) group (Fig. 1B). The 0.1 µg LPS/OVA (0.1LPS) group and Alum/OVA (EOS) group also showed significantly increase in Penh upon exposure to aerosolized methacholine compared with the control group. Meanwhile, from that of normal controls, the CFA group showed increase in Penh but did not reach statistical significance. However, the severity of lung histopathology in the CFA group is not similar to its AHR. The CFA group displayed more severe bronchiolar and perivascular inflammation infiltration as assessed by H&E staining than the other four groups (Fig. 1C and 1E). The inflammatory cell infiltration in the 0.1LPS group and 10LPS group was also more than that in the control group, but lower than that in the CFA group. We assessed mucin secretion in the airway of mice by using PAS staining and also observed similar results to H&E staining (Fig. 1D and F).

### The ability of CFA to induce neutrophil inflammation is stronger than that of LPS

**Fig. 2:**
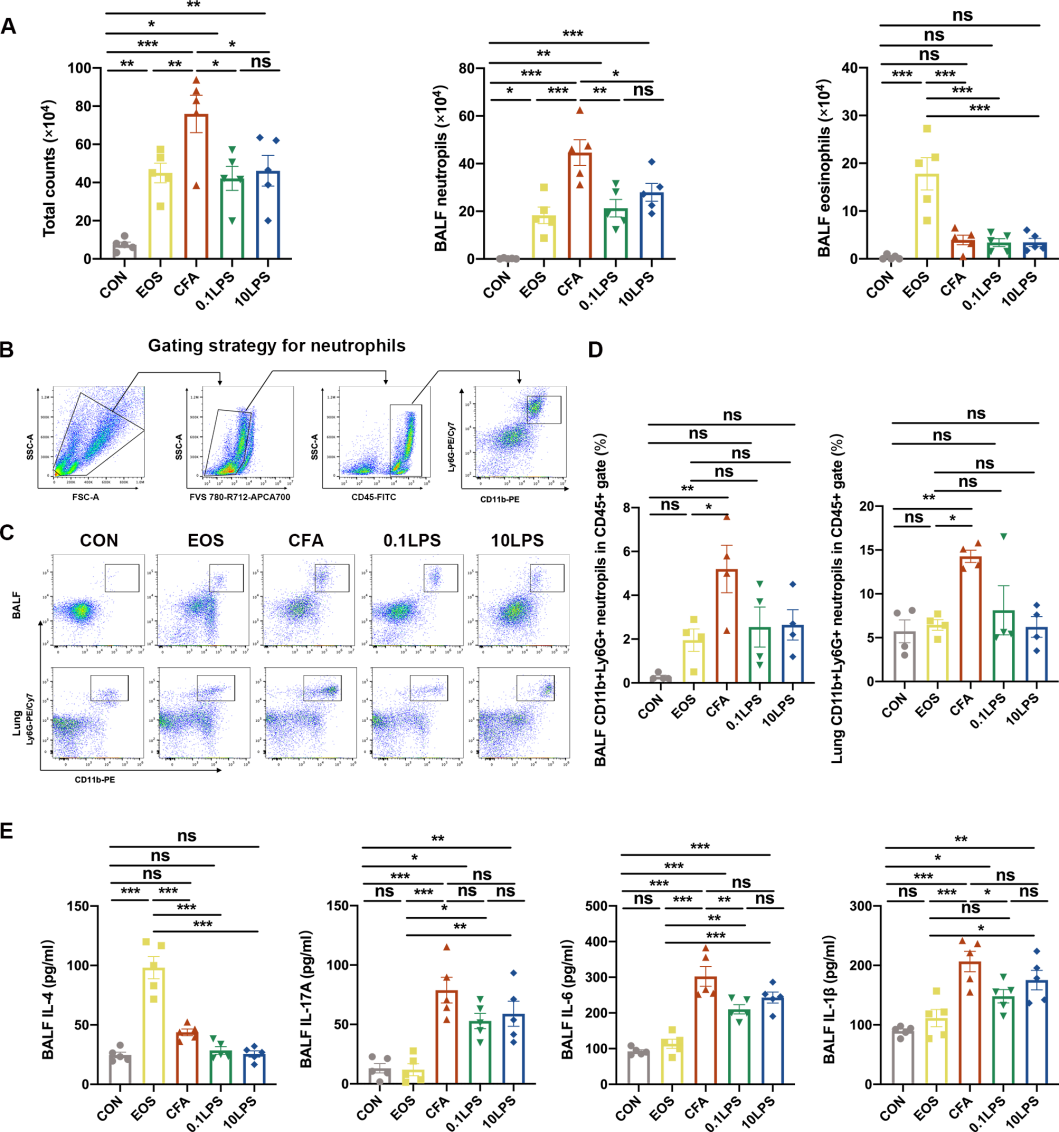
Neutrophil inflammatory infiltration was more pronounced in the CFA-induced neutrophil mouse models. (A) The number of total cells, eosinophils and neutrophils in BALF were quantified at 48 h after the last challenge. (B) Gating strategy for neutrophils is define as cells with the following characteristics: CD45(+)CD11b(+)Ly6G(+). (C) Neutrophil expression in CD45(+) leucocytes in BALF and lung tissue was determined by flow cytometry at 48 h after the last challenge. The representative images in each group are shown. (D) Quantification: the percentage of CD11b(+)Ly6G(+) neutrophils in the CD45(+) leucocytes gate of BALF and lung tissue by flow cytometry. (E) The cytokines interleukin (IL)-4, IL-17A, IL-6 and IL-1β levels in BALF were measured by enzyme-linked immunosorbent assay (ELISA). Data were shown as mean ± SEM; n = 5 in (A) and (E); n = 4 in (C) and (D). Significance between groups was calculated using one-way ANOVA with Tukey’s post hoc method. *p < 0.05, **p < 0.01 and ***p < 0.001

To further validate the inflammatory phenotype of the model, we also performed total and differential cell counts of BALF. Results demonstrated that the total number of cells and the percentage of neutrophils were significantly increased in the three groups of neutrophil models, particularly the CFA group (Fig. 2A). The percentage of eosinophils in the BALF of the three groups of neutrophil models had no significant difference compared with the control group, the EOS group of that were markedly increased (Fig. 2A). Moreover, for more precise evaluate the inflammatory phenotype of each group, we performed flow cytometry on cells of the BALF and single lung-cell suspensions of mice. Neutrophils was gated as viable CD45(+)CD11b(+)Ly6G(+) cells (Fig. 2B). Although the percentage of neutrophils in CD45(+) leukocytes increased in the BALF and single lung-cell suspensions of the three groups of neutrophil models compared to the control group, only the CFA group had a statistically significant difference (Fig. 2C and 2D). Similarly, only the percentage of neutrophils in CD45(+) leukocytes of the CFA group was statistically significant compared to the eosinophil group (Fig. 2 C and D). We next assessed the impact of different allergens on inflammatory cytokines in BALF. As a marker of Th2 polarization, IL-4 was obviously increased in the EOS group, while there was no significant change in the three neutrophil groups. Unlike that, the three neutrophil groups, especially the CFA group, had significantly higher IL-17 A, IL-6 and IL-1β expression than the control or EOS group (Fig. 2E).

### The neutrophil mouse models developed obvious NETosis

**Fig. 3:**
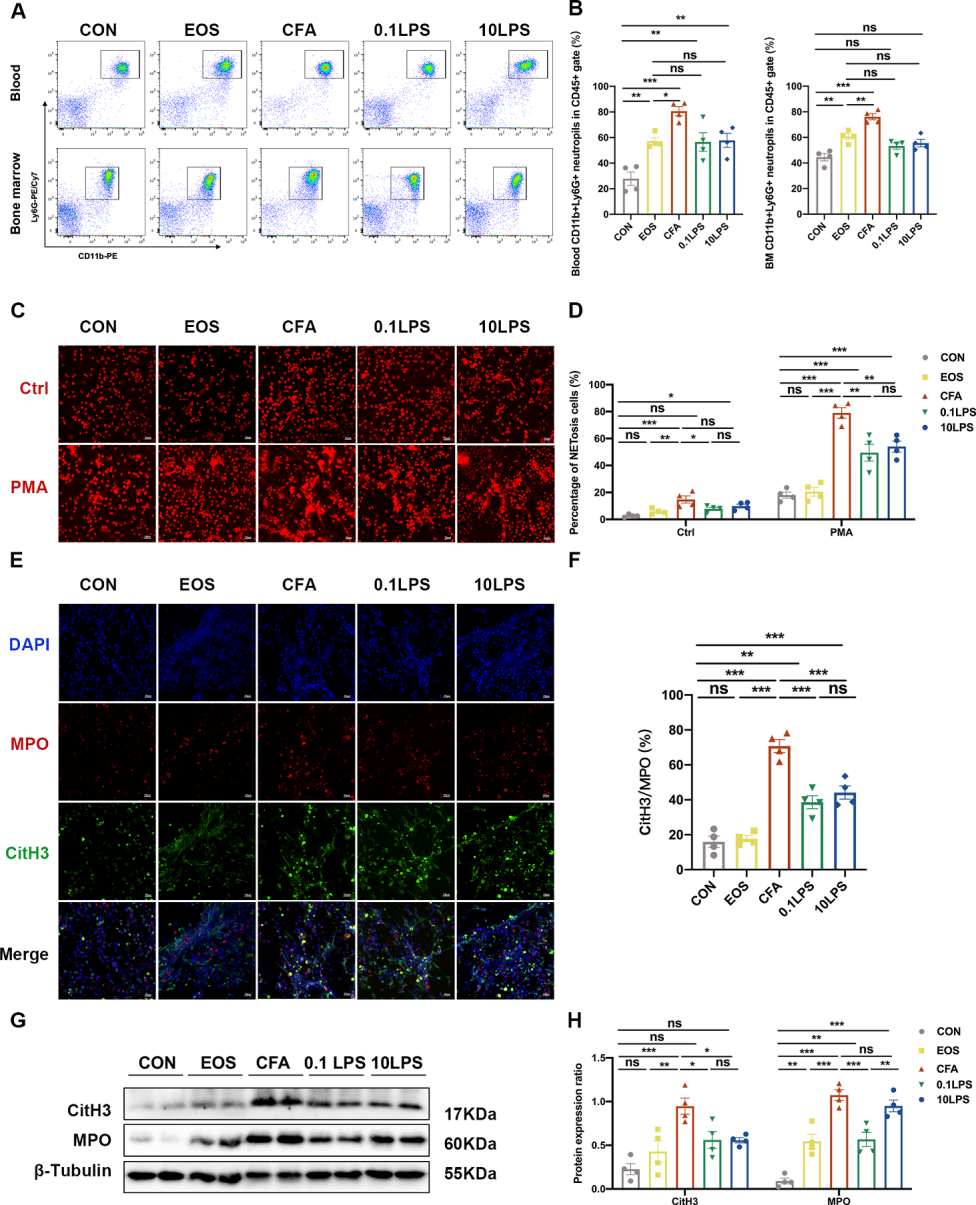
Abundant NETs occur in neutrophilic asthma models. (A) Neutrophil expression in CD45(+) leucocytes in mouse peripheral blood and bone marrow were determined by flow cytometry within 48 h after the last challenge. The representative images in each group are shown. (B) Quantification: the percentage of CD11b(+)Ly6G(+) neutrophils in the CD45(+) leucocytes gate of mouse peripheral blood and bone marrow by flow cytometry. (C) In vitro NET-formation assays with purified neutrophils of mouse bone marrow neutrophils stimulated with PMA (100 nM) or RPMI 1640 for 4 h. Then the neutrophils were stained PI and analyzed by immunofluorescence confocal microscopy. Representative immunofluorescence images of NETs, Scale bar = 20 μm. (D) Comparison of the percentage of NETosis cells in each group upon PMA stimulation and control (Ctrl) stimulation. (E) After stimulated with PMA, the neutrophils were stained for myeloperoxidase (MPO, red), citrullinated histone 3 (CitH3, green) and DAPI (nuclear staining, blue) and analyzed by immunofluorescence confocal microscopy. Representative immunofluorescence images of NETs, Scale bar = 20 μm. (F) Percentage of NETs area normalized to MPO positive signal in mouse bone marrow neutrophils after PMA stimulation. (G, H) Western blot was used to detect the levels of MPO and CitH3 protein in lung tissue of five groups of mice. Expression is relative to β-Tubulin. Cropped blots are shown, and supplementary Fig. S1 and S4 presents the full-length blots. Data were shown as mean ± SEM; n = 4. Significance between groups was calculated using one-way ANOVA with Tukey’s post hoc method. *p < 0.05, **p < 0.01 and ***p < 0.001

To further explore the mechanism of neutrophils in mouse model of neutrophilic asthma, we first assessed neutrophil infiltration in mouse peripheral blood and bone marrow by flow cytometry. Results revealed that the percentage of neutrophils in the CFA group in both peripheral blood and bone marrow were found to be highest among all five groups. Although the percentage of neutrophils in the 0.1LPS group and the 10LPS group increased significantly relative to the control group, it was not as significant as the CFA group. In the flow cytometry analysis of bone marrow cells, the percentage of CD11b(+)Ly6G(+) neutrophils in the 0.1LPS group and 10LPS group lower than that in the EOS group (Fig. 3A and 3B). As the first line of defense, neutrophils partially mediate host responses by establishing NETs [20]. PMA is a very powerful, but non-physiological stimulus for NET formation [18]. We next purified mouse bone marrow neutrophils and stimulated with PMA (100 nM) in vitro to see whether NETs were released. Confocal microscopy revealed that a large number of NETosis neutrophils and NETs were detected in all three neutrophil models, with the highest amount of that produced in the CFA group. There was a small number of NETs observed in the EOS group (Fig. 3C-F). We also found that only a minority of neutrophils developed NETosis in the absence of stimulation, whether isolated from the bone marrow of the neutrophil models or other models. In addition, we detected the expression levels of MPO and CitH3 using western blot in lung tissue of mice, and the results were consistent with that of immunofluorescence analysis (Fig. 3G and H).

### The neutrophilic asthma model induced by 0.1LPS and CFA combined with OVA exhibited significant AHR and severe airway infiltration

**Fig. 4:**
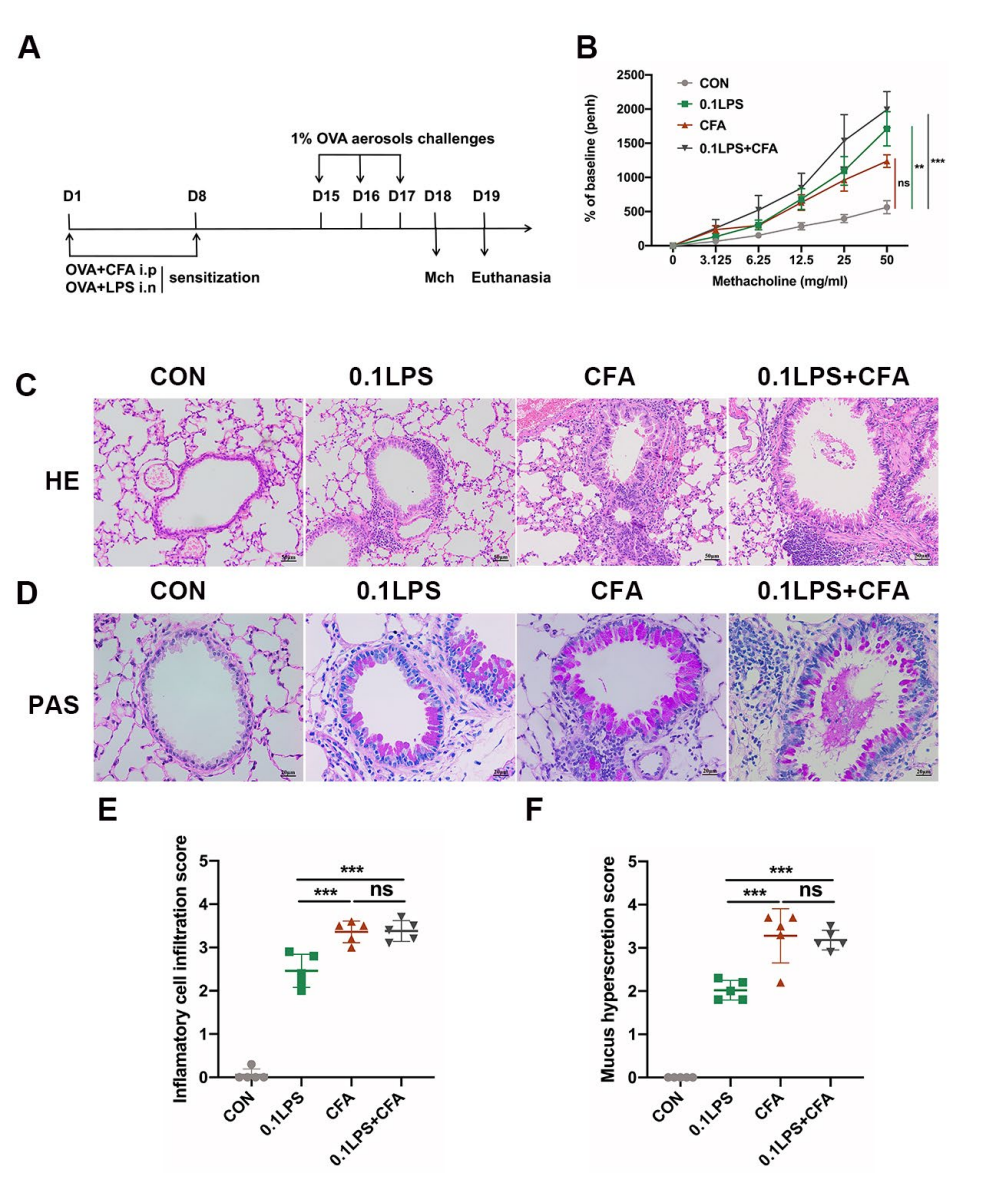
The 0.1LPS and CFA combined with OVA-induced neutrophilic asthma model exhibits significant AHR and severe airway infiltration. (A) Schematic diagram of the experiment. (B) The AHR was measured 24 h after the last challenge, enhanced pause (Penh) values were used to assess impaired lung function. (C) Hematoxylin and eosin (H&E) staining of lung tissue. Scale bar = 50 μm. (D) Paraffin acid-Schiff (PAS) staining of lung tissue. Scale bar = 20 μm. (E) Quantification of inflammation infiltration score of the H&E staining. (F) Quantification of mucus-producing goblet cells of the PAS staining. Data were shown as mean ± SEM; n = 5. Significance between groups was calculated using one-way ANOVA with Tukey’s post hoc method. *p < 0.05, **p < 0.01 and ***p < 0.001

We summarize the above results and conclude that the CFA group is a relatively more suitable model of neutrophilic asthma, although the AHR of this model is not significant. To overcome this, we attempted to establish a new murine model combining the sensitization methods of the 0.1LPS group and the CFA group (0.1LPS + CFA group) (Fig. 4A). Result shown that the 0.1LPS + CFA group exhibited higher AHR in response to methacholine compared with the 0.1LPS or CFA group, although the differences were not statistically significant (Fig. 4B). Similar to the CFA group, lung histopathological examination in 0.1LPS + CFA group showed obvious inflammatory cells infiltration and airway mucus secretion, some airways showed local airway mucus obstruction (Fig. 4C-F). These results seem to indicate that the 0.1LPS + CFA group serve as a suitable model of mouse to study the pathogenesis of the neutrophilic asthma.

### CFA combined with LPS induced strong neutrophilic inflammation and secretion of proinflammatory factors

**Fig. 5:**
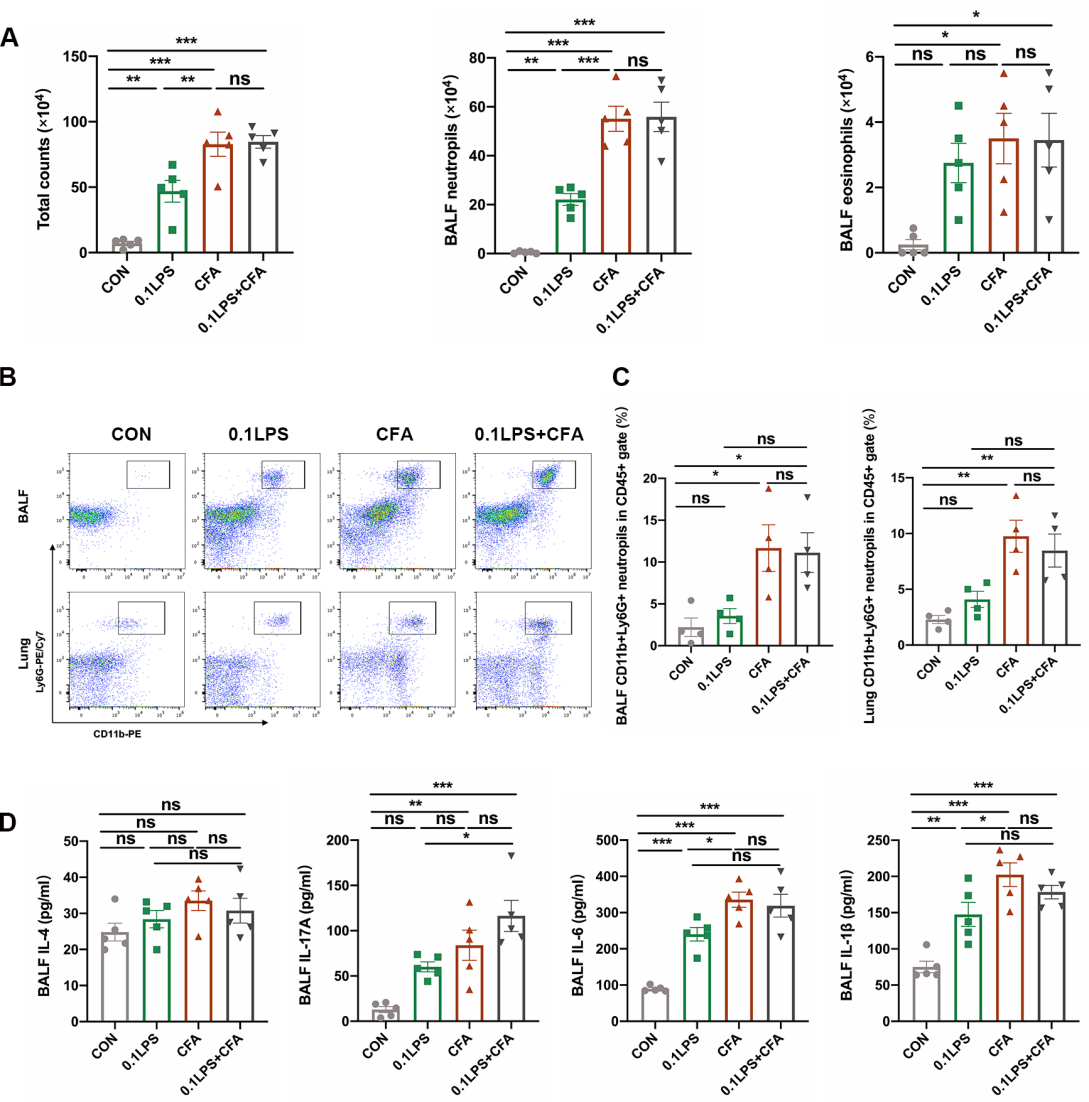
Significant neutrophilic inflammation and secretion of proinflammatory factors were observed in CFA combined with LPS induced mouse model. (A) Quantification of total cell counts and differential cell counts (eosinophils and neutrophils) in BALF at 48 h after the last challenge. (B) The percentage of CD11b(+)Ly6G(+) neutrophils in CD45(+) leucocytes in BALF and lung tissue was determined by flow cytometry at 48 h after the last challenge. The representative images of each group are shown. (C) Statistical analysis of the above data shown by flow cytometry. (D) Cytokine (IL-4, IL-6 and IL-1β) concentrations in BALF were quantified by enzyme-linked immunosorbent assay (ELISA). Data were shown as mean ± SEM; n = 5 in (A) and (D); n = 4 mice in (B) and (C). Significance between groups was calculated using one-way ANOVA with Tukey’s post hoc method. *p < 0.05, **p < 0.01 and ***p < 0.001

Immediately after, we performed BALF and lung tissue cells analysis. The total number of BALF in the 0.1LPS + CFA group was significantly higher than that in the control and 0.1LPS groups, and there was no significant difference in total number of BALF between the 0.1LPS + CFA and CFA groups (Fig. 5A). Similar results were observed between the groups in the percentage of neutrophils (Fig. 5A). The percentage of eosinophils in the BALF of the three neutrophil groups had no significant difference compared with each other, while higher than that in the control group (Fig. 5A). Likewise, we performed flow cytometry on BALF cells and single lung-cell suspensions to better understand our newly established neutrophil model. The percentage of neutrophils in CD45(+) leukocytes in the BALF and single lung-cell suspensions in both 0.1LPS + CFA and CFA groups was significantly increased, and the difference was statistically significant (Fig. 5B and C). The 0.1LPS group also shown more percentage of neutrophils in CD45(+) leukocytes compared with the control group, but no statistical significance was achieved (Fig. 5B and C). Next, we quantification the expression of inflammatory cytokines in BALF. As expected, the expression of IL-17A, IL-6 and IL-1β in the 0.1LPS + CFA group and other two neutrophil groups were strongly increased compared with the control group. However, the expression of IL-4 was not significantly different in all four groups (Fig. 5D).

### The OVA/CFA/LPS-induced mice model developed obvious NETosis

**Fig. 6:**
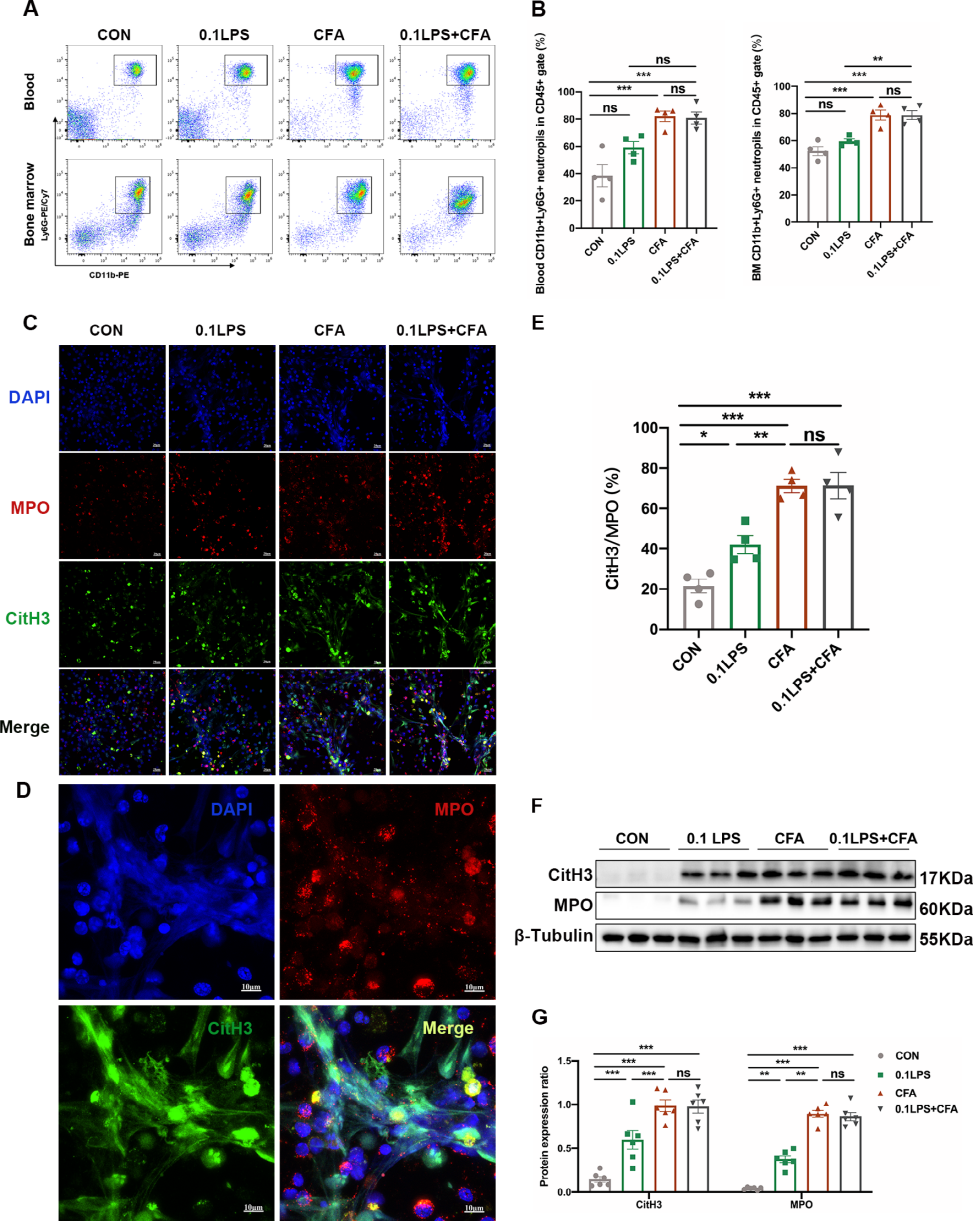
Neutrophilic mouse model induced by CFA combined with LPS showed enhanced NETs formation capacity. (A) At 48 h after the last challenge, the percentage of neutrophil populations in CD45(+) leukocytes in mouse peripheral blood and bone marrow were determined by flow cytometry. The representative images in each group are shown. (B) Statistical analysis of the percentage of CD11b(+)Ly6G(+) neutrophils in CD45(+) leukocyte gate of mouse peripheral blood and bone marrow by flow cytometry. (C) Neutrophils were purified from mouse bone marrow and stimulated with PMA (100 nM) or vehicle control for 4 h. Then, neutrophils were stained for myeloperoxidase (MPO, red), citrullinated histone 3 (CitH3, green), and DNA (DAPI, blue) and confocal by immunofluorescence microscope for analysis. Representative images of NETs immunofluorescence. Scale bar = 20 μm. (D) Percentage of NETs area normalized to MPO positive signal in mouse bone marrow neutrophils after PMA stimulation. (E) Representative z-axis images of the NETs immunofluorescence in 0.1LPS + CFA group. Scale bar = 10 μm. (F, G) Western blot analysis the protein expression level of MPO and CitH3 in lung tissue of four groups of mice. Expression is relative to β-Tubulin. Cropped blots are shown, and supplementary Fig. S2 and S5 presents the full-length blots. Data were shown as mean ± SEM; n = 4 or 6. Significance between groups was calculated using one-way ANOVA with Tukey’s post hoc method. *p < 0.05, **p < 0.01 and ***p < 0.001

Likewise, to investigate whether neutrophils in the 0.1LPS + CFA group asthma mouse model also have a similar mechanism to the neutrophil groups described above, we also evaluated neutrophil infiltration in mouse peripheral blood and bone marrow by flow cytometry. Results demonstrate that the percentage of neutrophils in peripheral blood and bone marrow in the 0.1LPS + CFA group was significantly higher than that in the control group, which was comparable to the CFA group. The percentage of neutrophils in the 0.1LPS group was increased compared with the control group, but there was no statistical difference (Fig. 6A and B). Confocal microscopy demonstrated that NETs co-localized with MPO, citH3 and DAPI were detected in all three neutrophil models after PMA stimulation, while the 0.1LPS + CFA group and CFA group could observe the most significant NETs release (Fig. 6C-E). Western blot also showed that the protein expression levels of MPO and CitH3 in the lung tissue of mice in each group were consistent with the results of immunofluorescence (Fig. 6F and G).

### DNase I or CI-amidine administration protects mice of neutrophilic asthma model

**Fig. 7:**
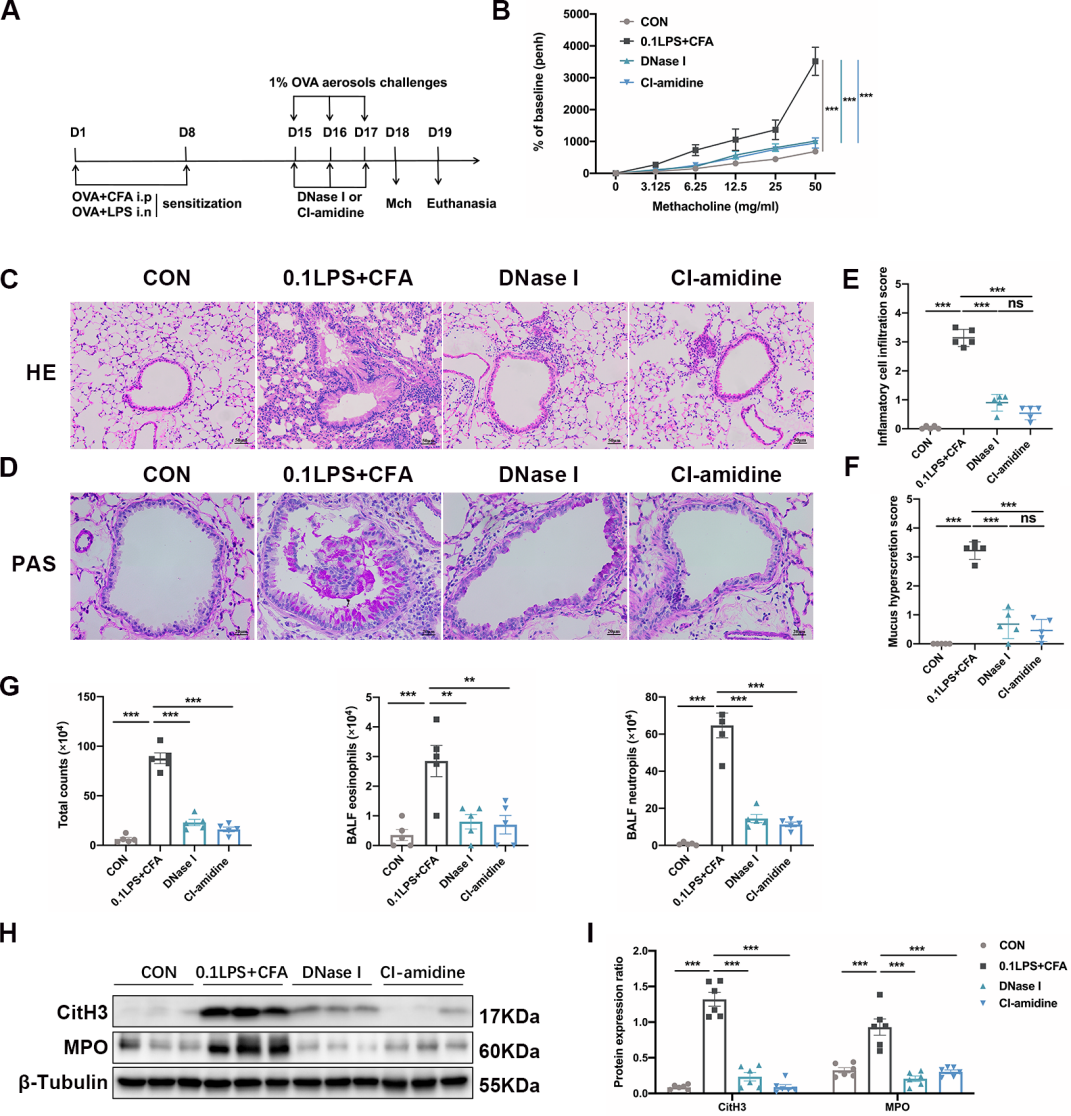
DNase I or CI-amidine administration reduced airway hyperresponsiveness and alleviated airway inflammation (A) Experimental assay schematic for in vivo experiments. (B) AHR was measured 24 h after the last challenge, enhanced pause (Penh) values were used as an indicator of lung function. (C) Hematoxylin and eosin (H&E) staining of lung tissue. Scale bar = 50 μm. (D) Paraffin acid-Schiff (PAS) staining of lung. Scale bar = 20 μm. (E) Quantification of inflammation infiltration score of the H&E staining. (F) Quantification of mucus-producing goblet cells of the PAS staining. (G) The total number of cells and the differential number of cells (eosinophils and neutrophils) were quantified 48 h after the last challenge in the BALF. (H, I) Western blot analysis to measure MPO and CitH3 protein expression level in lung tissue of four groups of mice. Expression is relative to β-Tubulin. Cropped blots are shown, and supplementary Fig. S3 and S6 presents the full-length blots. Data were shown as mean ± SEM; n = 4–6. Significance between groups was calculated using one-way ANOVA with Tukey’s post hoc method. *p < 0.05, **p < 0.01 and ***p < 0.001

To determine whether the presence of NETs contributes to the pathogenesis of neutrophilic asthma model, we treated mice with either DNase I or CI-amidine before each challenge (Fig. 7 A). Previous studies have shown that DNase I can break down NETs [21], while activation of PAD4 is a major driver of NETs formation, and CI-amidine is an irreversible PAD4 inhibitor [22, 23]. Here, we found that DNase I or CI-amidine treatment significantly reduced airway hyperresponsiveness (Fig. 7B) and alleviated airway inflammation in the 0.1LPS + CFA group (Fig. 7C and E), PAS staining showed that airway mucus obstruction disappeared (Fig. 7D and F). Analysis of inflammatory cells in BALF also indicated that both the total number of cells and the percentage of neutrophils were significantly reduced by DNase I or CI-amidine treatment (Fig. 7G). Western blot analysis of lung tissue showed that the expression of MPO and CitH3 were significantly reduced in the OVA/CFA/0.1 LPS + DNase I (DNase I) group or the OVA/CFA/0.1 LPS + CI-amidine (CI-amidine) group (Fig. 7 H and I).

## Discussion

Asthma is a heterogeneous disease with multiple phenotypes and endotypes [24, 25]. Approximately 5-25% of asthmatics suffer from severe asthma and do not respond to existing medical treatment, those people account for 50-80% of all asthma-related health care costs [26]. Neutrophilic airway inflammation is one of the main features of severe asthma. As of today, although there are many studies on neutrophilic asthma, the pathogenesis of neutrophilic asthma is poorly understood, and there are still many difficulties in treatment.

OVA is a classic allergen for establishing allergic asthmatic mouse models, which usually requires co-sensitization with appropriate adjuvants [27]. The type of OVA-induced airway inflammation varies depending on the adjuvant. OVA/CFA or OVA/LPS is often used to develop neutrophilic asthma mouse models by investigators. CFA is known as a powerful inflammatory stimulus and widely used experimental models of arthritis [28]. In recent years, many studies have successfully established neutrophilic asthma model using CFA combined with OVA [8, 10, 29]. This model have higher degrees of airway neutrophilic inflammation, however airway responsiveness is not significantly increased [30]. This could be explained by the fact that despite the association between AHR and airway inflammation, there is evidence that AHR can occur independently of inflammation [31–33]. A similar phenomenon was also found in our study, although Penh in the CFA group increased compared with the control group, it was lower than other neutrophil groups or even the eosinophil group.

LPS is a major component of the outer membrane of gram-negative bacteria, which stimulates the innate immune response to inflammation [34]. The use of LPS alone cannot establish a mouse model of asthma, and it acts as an adjuvant in the asthma model. Neutrophilic airway inflammation and airway hyperresponsiveness can be induced when LPS is used in combination with OVA [9, 35]. However, in asthma models, the role of LPS remains controversial. LPS is present at high levels in air and dust, some studies indicated that the extent of LPS exposure negatively correlates with the risk of developing asthma [36], while others held the opposite opinion that LPS in the environment probably plays an important role in the occurrence of asthma exacerbations and insensitive responses to corticosteroids in humans [37]. According to Eisenbarth et al. [38], low dose LPS promote Th2 immunity, whereas high doses promote Th1 responses. This view is also supported by a recent study showing that exposure to low-dose LPS (0.1 µg) in BALB/c mice enhanced type 2 allergic asthma, whereas starting with a higher dose of LPS (10 µg) had no significant effect [39]. However, there is also study using 50 µg OVA with 0.1 µg LPS sensitization combined with OVA challenge to successfully establish Th17-dependent neutrophilic airway inflammation [9]. We speculate that different effects of LPS in various studies in asthma are due to differences in time and dose. Following the studies described above, we established the OVA/low-dose (0.1 µg) LPS and OVA/high-dose (10 µg) LPS models. Our work revealed that both the 0.1LPS group and 10LPS group showed significant airway hyperresponsiveness, much higher than the CFA group, while the inflammatory cell infiltration in the 0.1LPS group and 10LPS group was lower than that in the CFA group. Based on the above conclusions, with consideration of high doses of LPS may cause acute lung injury, we combined OVA/CFA/0.1LPS to establish a mouse model and subsequent experimental validations. The results showed that the 0.1LPS + CFA group exhibited marked airway hyperresponsiveness and airway inflammation.

NETs are composed of histones, neutrophil elastase (NE), MPO and double-stranded (ds) DNA [40, 41]. NETs are important in antibacterial defense, helping to limit systemic infection and maintain host defenses against fungal pathogens [42]. When the body was stimulated by pathogenic or chemical stimuli, the neutrophils use degranulation, phagocytosis, and the production of NETs to control initial infections [43]. However, with our knowledge about NETs have been greatly expanded in recent years, accumulating evidence indicated that NETs are considered to be a double-edged sword in lung disease. Infectious and noninfectious pulmonary diseases led to large-scale neutrophil infiltration into the lungs, and activated neutrophils release substantial amounts of NETs [44–46]. However, excessive NETs generation in noninfectious settings could be damaging for the tissue/organ, excessive NETs formation increases mucus viscosity and fills the lungs, impairing lung function [44, 47]. In the neutrophilic asthma models of our study, the group with more severe airway inflammation also produced more NETs. In the pathological staining of the 0.1LPS + CFA group, we could even see a large amount of mucus occluded the lumen. We speculate that this is related to the oversecretion of NETs. It has been shown that BALF from patients with severe asthma had detectable NETs that were positively correlated with IL-17 levels [48]. IL-1β is a proinflammatory cytokine central to the inflammatory response driven by the IL-6 signaling pathway [49]. Proinflammatory cytokines such as IL-1β, IL-6 are up-regulated when neutrophils undergo NETosis [50]. Also in our work, we observed increased expression of IL17A, IL-1β and IL-6 in the neutrophilic asthma models.

We validated our results by increasing NETs degradation or decreasing NETs generation to determine that the presence of NETs exacerbated disease in neutrophilic asthma model. In our previous experiments, we found that airway mucus secretion and airway responsiveness were higher in the 0.1LPS + CFA group than that in the other groups. After treated with DNase I, the airway hyperreactivity and mucus production of the 0.1LPS + CFA group, however, were almost completely eliminated. It has also previously been shown that airway mucus embolism in asthmatic patients can achieve complete lysis within minutes after administration of recombinant human DNase [51]. It now appears that recombinant human DNase can relieve airway mucus embolism, possibly by degrading airway NETs. Activation of PAD4 is likely a major driver of NETosis, and histone citrullination catalyzed by PAD4 appears to be a critical step in NETosis [22, 52]. Similarly, we demonstrate that a PAD4 inhibitor, CI-amidine, can significantly alleviate airway inflammation and airway hyperresponsiveness in neutrophilic asthma model. Both above-mentioned results confirmed NETs is critical to the pathogenesis of neutrophilic asthma model. In addition, we found that DNase I and CI-amidine had no significant impact on AHR of EOS mice (Fig S7). This can be explained as follows: DNase I and CI-amidine play a role in neutrophilic asthma model mainly because they can increase NETs degradation or decrease NETs generation. For NETs that are not highly expressed in the EOS group, DNase I and CI-amidine did not work.

However, the current study did have some shortcomings as well. How NETs is regulated in neutrophilic asthma remains unknown. Meanwhile, we also have recognized our study lacks the support of clinical correlative data. The issues mentioned above will require further study.

## Conclusion

In conclusion, our findings identify a novel mouse model of neutrophilic asthma induced by OVA, CFA and LPS. This kind of neutrophilic asthma model is phenotypically more comprehensive, and characterized by AHR, massive infiltration of neutrophils and production of non-Th2 cytokines. Our data also suggest that a large number of NETs are generated in vivo and in vitro in the neutrophilic asthma models, reducing NETs can reverse those changes mentioned above. These findings provide further insight into the asthma model studies and pathogenesis of neutrophilic asthma, targeting NETs is a novel strategy that may be effective for treating neutrophilic asthma in the future.

## Electronic supplementary material

Below is the link to the electronic supplementary material.

Supplementary Material 1
Fig. S1. Full length blots of Fig. 3G.
Fig. S2. Full length blots of Fig. 6 F.
Fig. S3. Full length blots of Fig. 7 H.
Fig. S4. Additional bands used for statistics in Fig. 3 H.
Fig. S5. Additional bands used for statistics in Fig. 6G.
Fig. S6. Additional bands used for statistics in Fig. 7I.

## Data Availability

The datasets used or analyzed during the current study are available from the corresponding author on reasonable request.

## List of abbreviations

AHR: Airway hyperresponsiveness.
OVA: Ovalbumin.
Alum: Aluminum.
HDM: House dust mite.
CFA: Complete Freund’s adjuvant.
LPS: Lipopolysaccharide.
NETs: Neutrophil extracellular traps.
SPF: Specific pathogen-free.
Mch: Methacholine.
BALF: Bronchoalveolar lavage fluid.
IL: Interleukin.
ELISA: Enzyme-linked immunosorbent assay.
HE: Hematoxylin and eosin.
PAS: Paraffin acid-Schiff.
RBC: Red blood cell.
PMA: Phorbol 12-myristate 13-acetate.
PFA: Paraformaldehyde.
RT: Room temperature.
PBS: Phosphate-buffered saline.
BSA: Bovine serum albumin.
CitH3: Citrullinated histone 3.
MPO: Myeloperoxidase.
DAPI: 4,6-diamidino-2-phenylindole.
SDS-PAGE: Sulfatepolyacrylamide gel electrophoresis.
PVDF: Transferred onto polyvinylidene fluoride.
SEM: Standard errors of measurement.
