# Supplementary material for: Neutrophil activation and NETosis are the predominant drivers of airway inflammation in an OVA/CFA/LPS induced murine model
Source: Respir Res. 2022 Oct 21;23:289. doi: 10.1186/s12931-022-02209-0 (PMC9587569; doi:10.1186/s12931-022-02209-0)
Supplement: Supplementary file 1 — Supplementary Material 1 Fig. S1. Full length blots of Fig. 3G. Fig. S2. Full length blots of Fig. 6 F. Fig. S3. Full length blots of Fig. 7 H. Fig. S4. Additional bands used for statistics in Fig. 3 H. Fig. S5. Additional bands used for statistics in Fig. 6G. Fig. S6. Additional bands used for statistics in Fig. 7I. [file 12931_2022_2209_MOESM1_ESM.docx]

**Neutrophil activation and NETosis are the predominant drivers of airway inflammation in an OVA/CFA/LPS induced murine model**

Mengling Xia^1^, Fei Xu^1^, Hangqi Ni^1^, Qing Wang^1^, Ruhui Zhang^1^, Yafang Lou^2^* and Jianying Zhou^1^*

These authors contributed equally: Mengling Xia, Fei Xu

* Corresponding authors.

Department of Respiratory Disease, Thoracic Disease Center, The First Affiliated Hospital, College of Medicine, Zhejiang University, No. 79, Qingchun Road, Hangzhou 310003, China.

E-mail address: [zjyhz@zju.edu.cn](mailto:zjyhz@zju.edu.cn)

Department of Respiratory Medicine, Hangzhou Hospital of Traditional Chinese Medicine, No. 453, Tiyuchang Road, Hangzhou 310013, China.

E-mail address: [louyafang18@126.com](mailto:louyafang18@126.com)

**Supplementary materials**

**a**


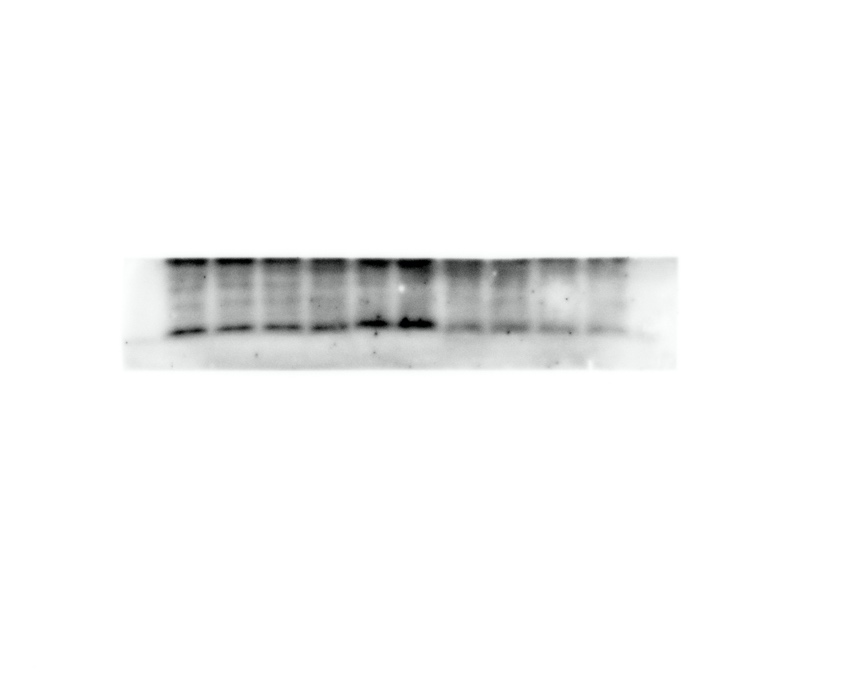


**b**


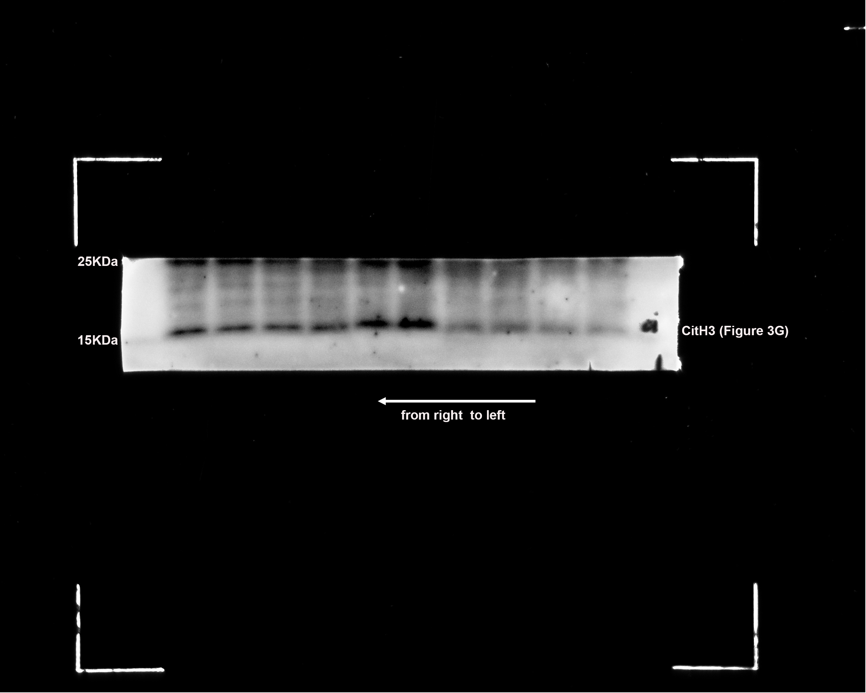


**c**


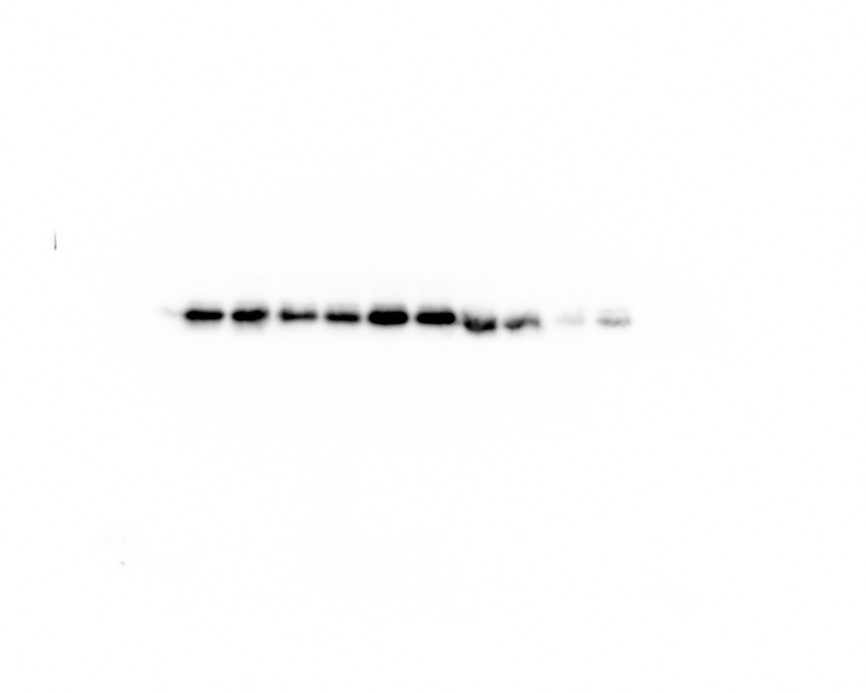


**d**


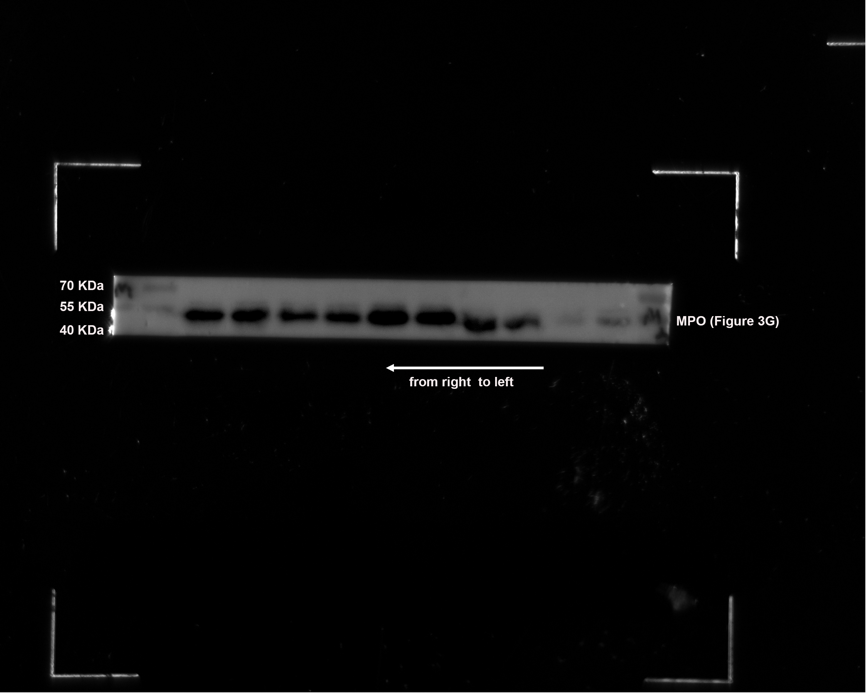


**e**


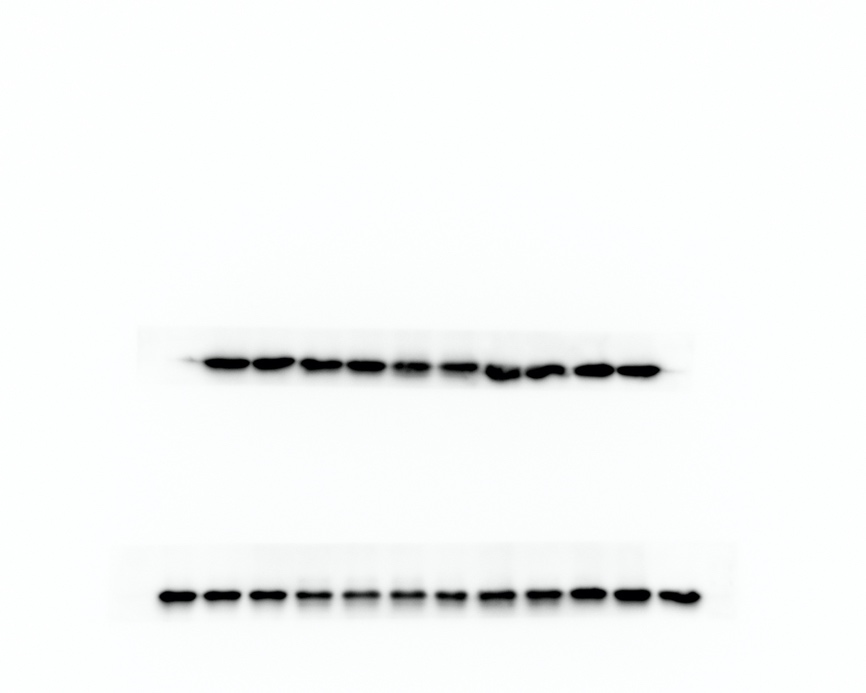


**f**


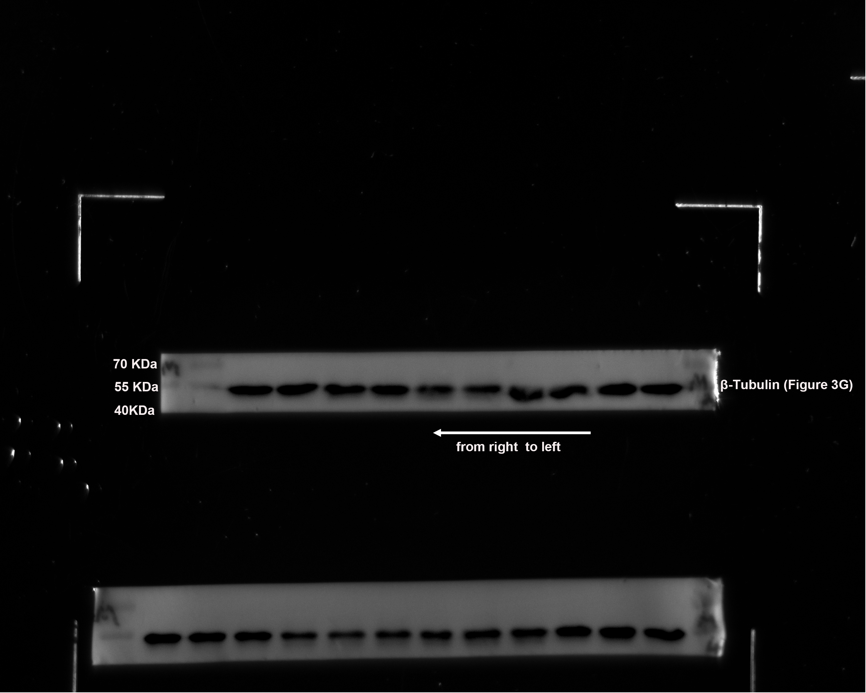


Supplementary Figure 1. Full length blots of Figure 3G. Proteins were separated by 10% SDS-PAGE. (a) (b) for CitH3; (c) (d) for MPO; (e) (f) for β-Tubulin (the upper band). MPO and β-Tubulin are the same membrane that has undergone two different primary antibody incubations respectively. All original images are flipped horizontally in Figure 3G to correspond to the order of the other parts. The above bands are also used for statistics in Figure 3H.

**a**


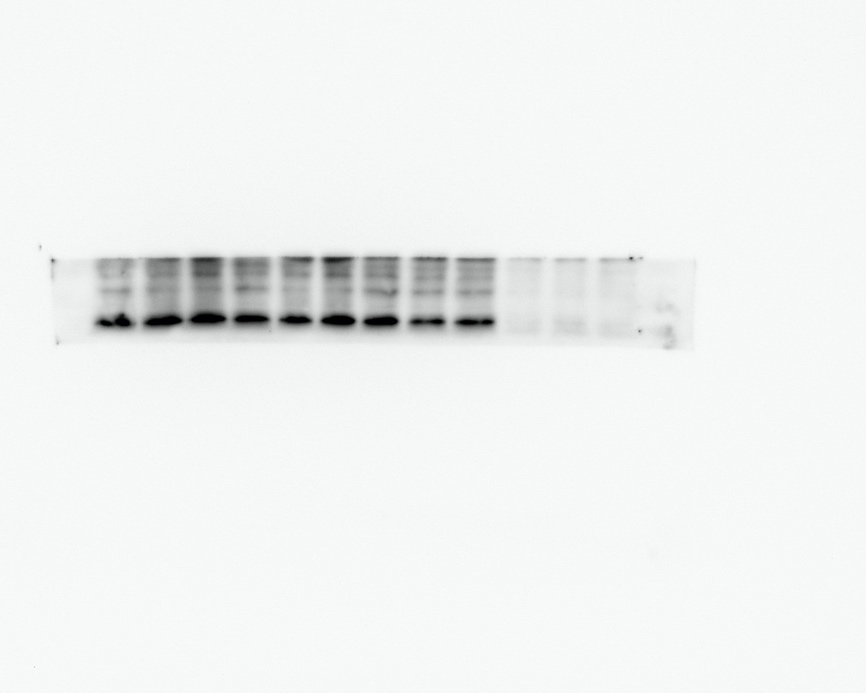


**b**


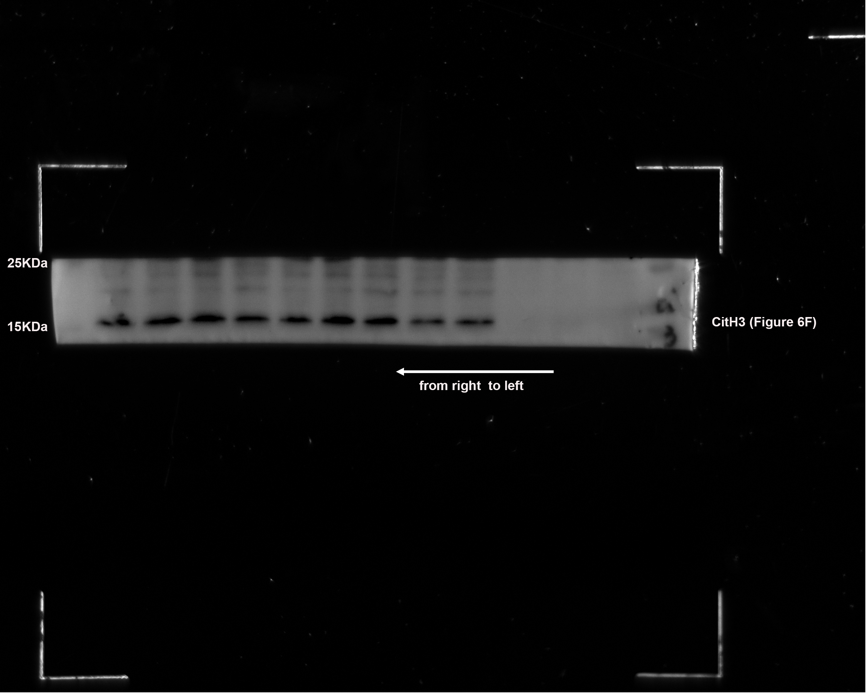


**c**


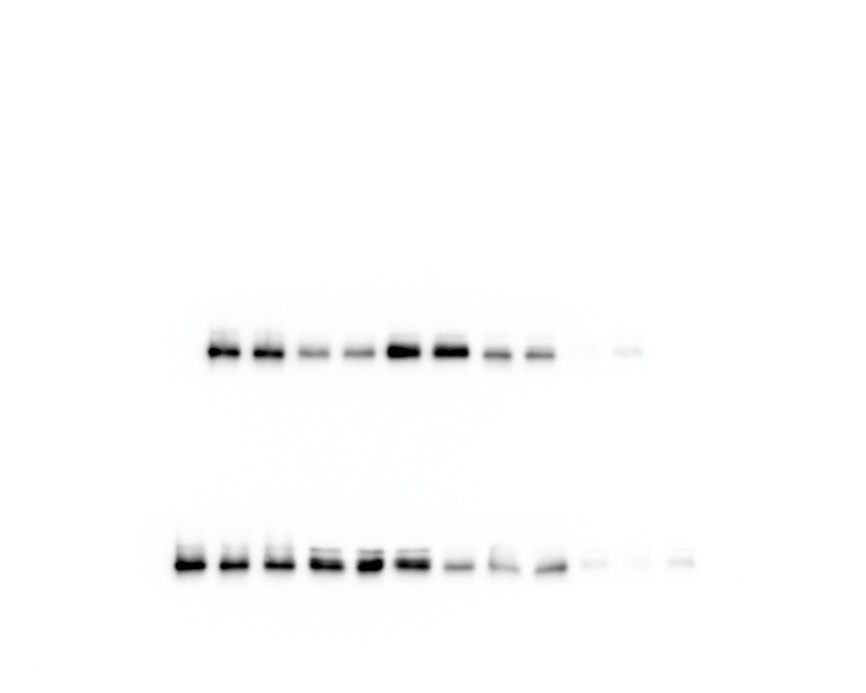


**d**

**
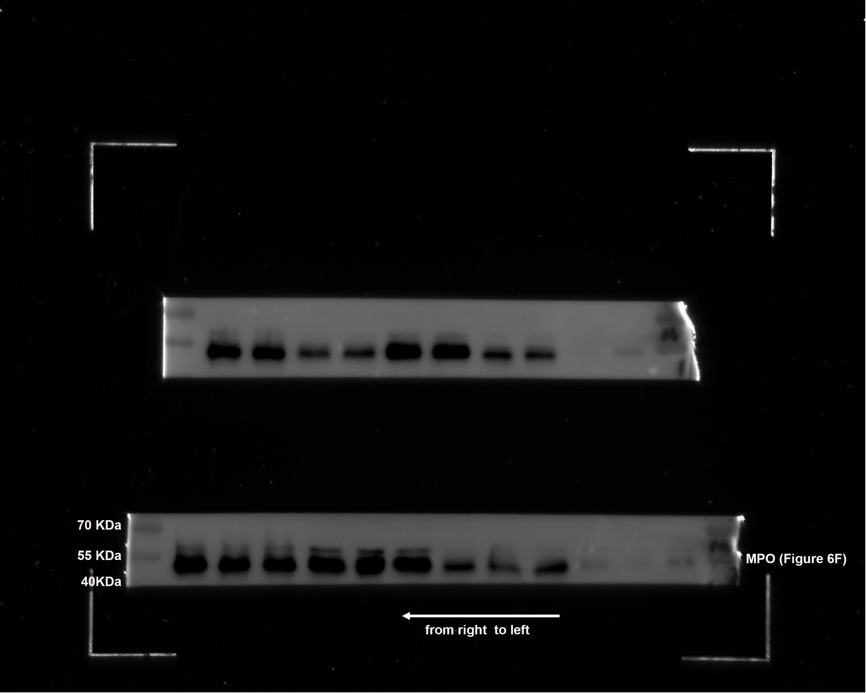
**

**e**

**
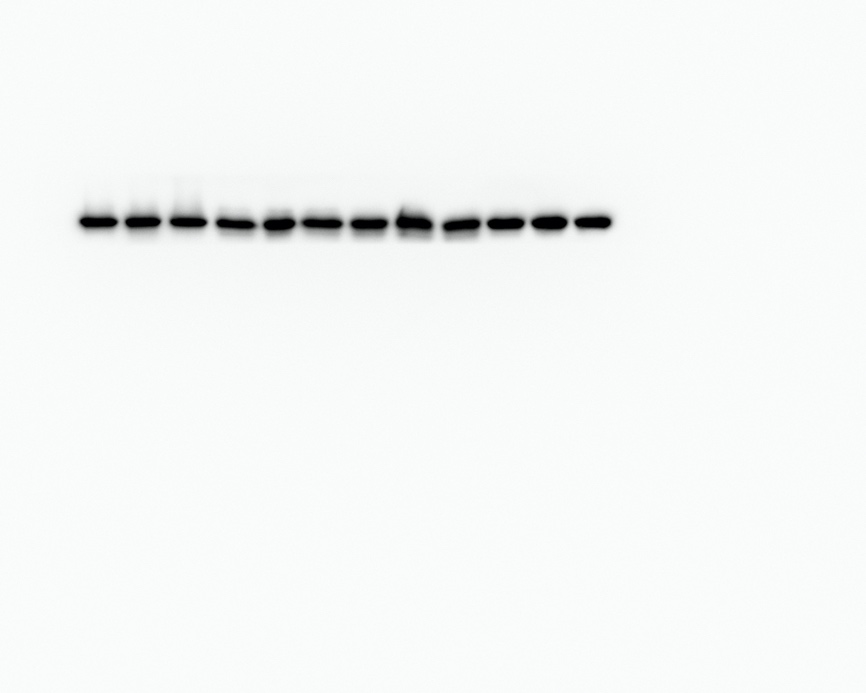
**

**f**

**
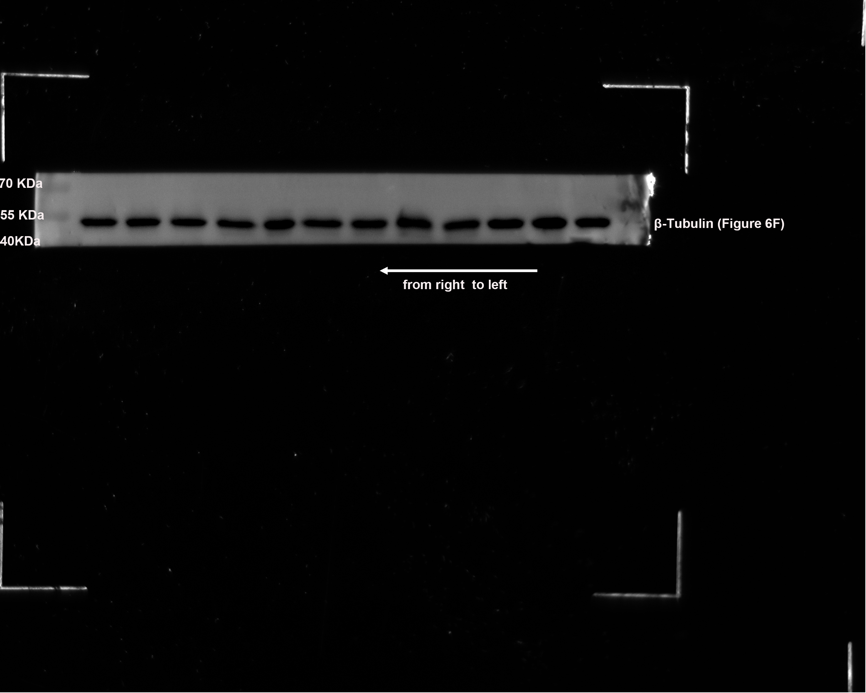
**

Supplementary Figure 2. Full length blots of Figure 6F. Proteins were separated by 10% SDS-PAGE. (a) (b) for CitH3; (c) (d) for MPO (the lower band); (e) (f) for β-Tubulin. MPO and β-Tubulin are the same membrane that has undergone two different primary antibody incubations respectively. All original images are flipped horizontally in Figure 6F to correspond to the order of the other parts. The above bands are also used for statistics in Figure 6G.

**a**


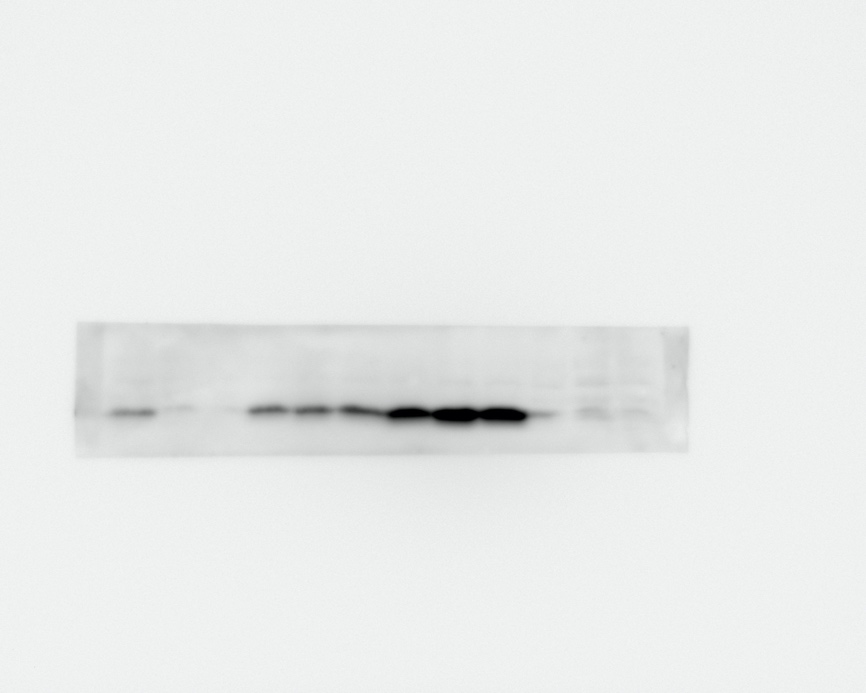


**b**


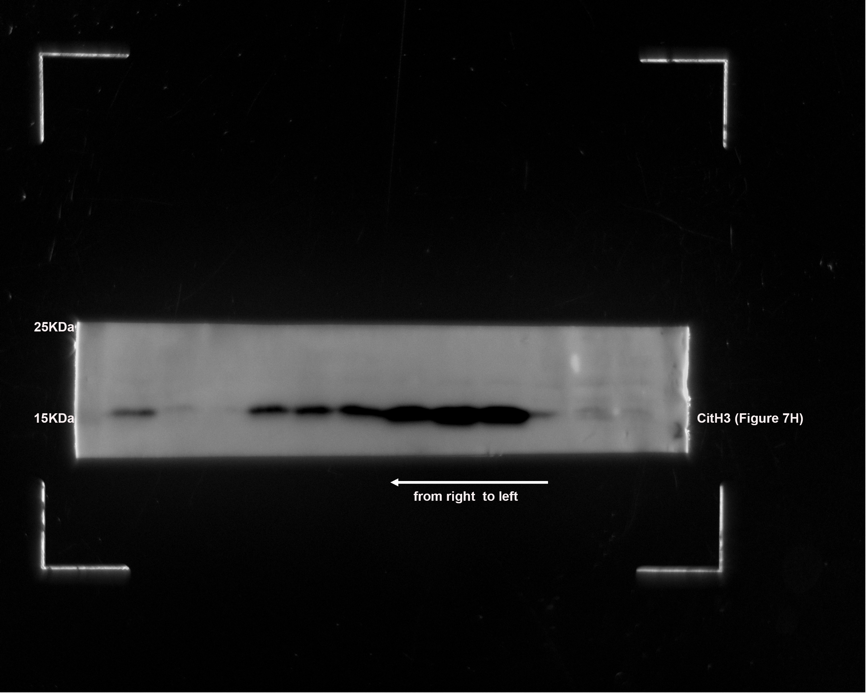


**c**

**
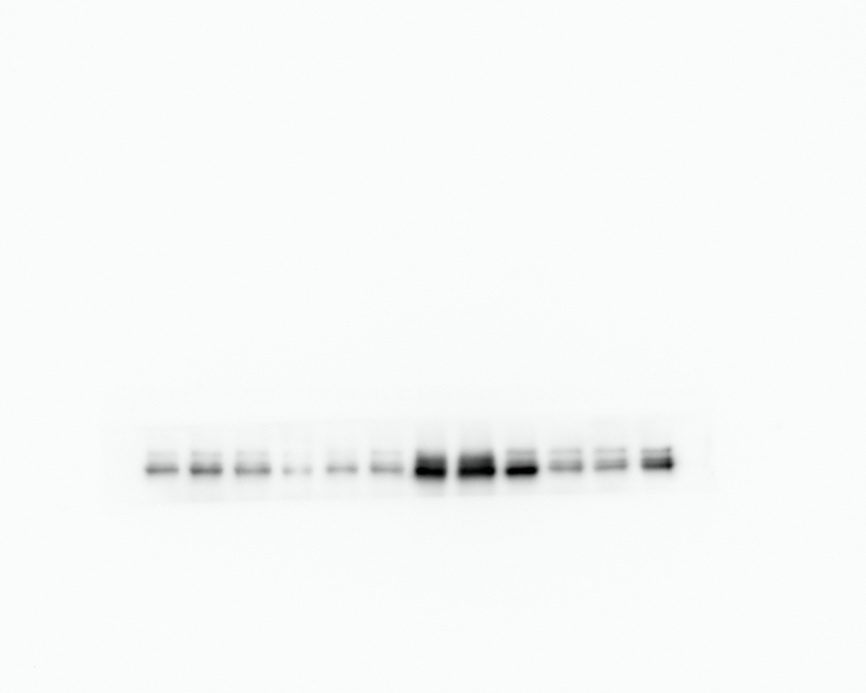
**

**d**

**
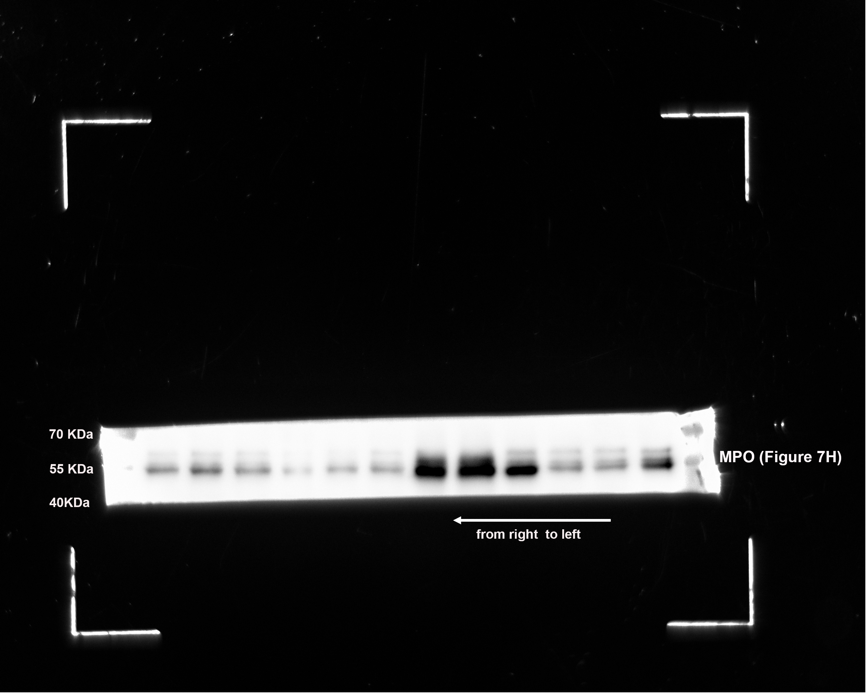
**

**e**

**
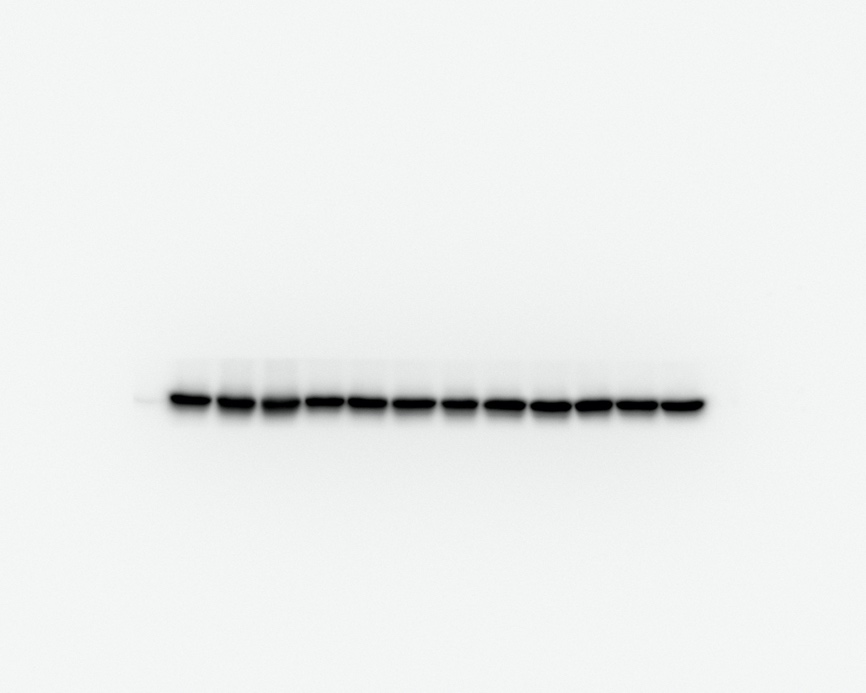
**

**f**

**
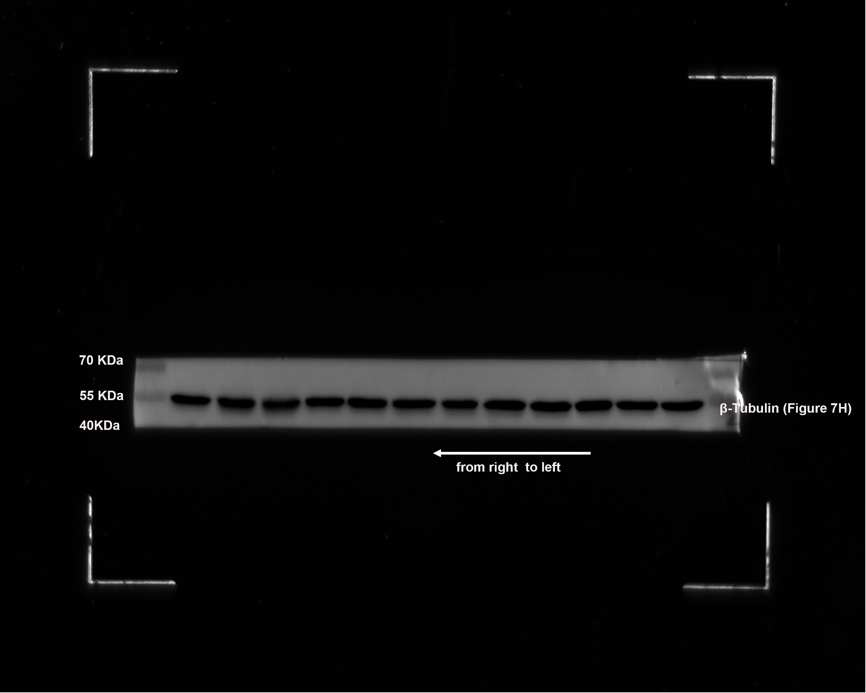
**

Supplementary Figure 3. Full length blots of Figure 7H. Proteins were separated by 12% SDS-PAGE. (a) (b) for CitH3; (c) (d) for MPO; (e) (f) for β-Tubulin. All original images are flipped horizontally in Figure 7H to correspond to the order of the other parts. The above bands are also used for statistics in Figure 7I.

**a**


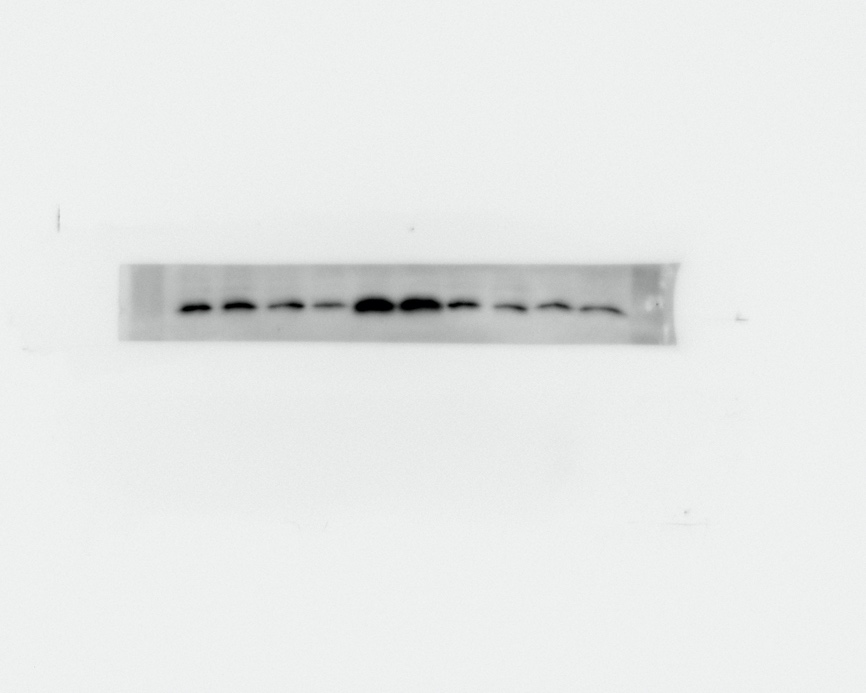


**b**


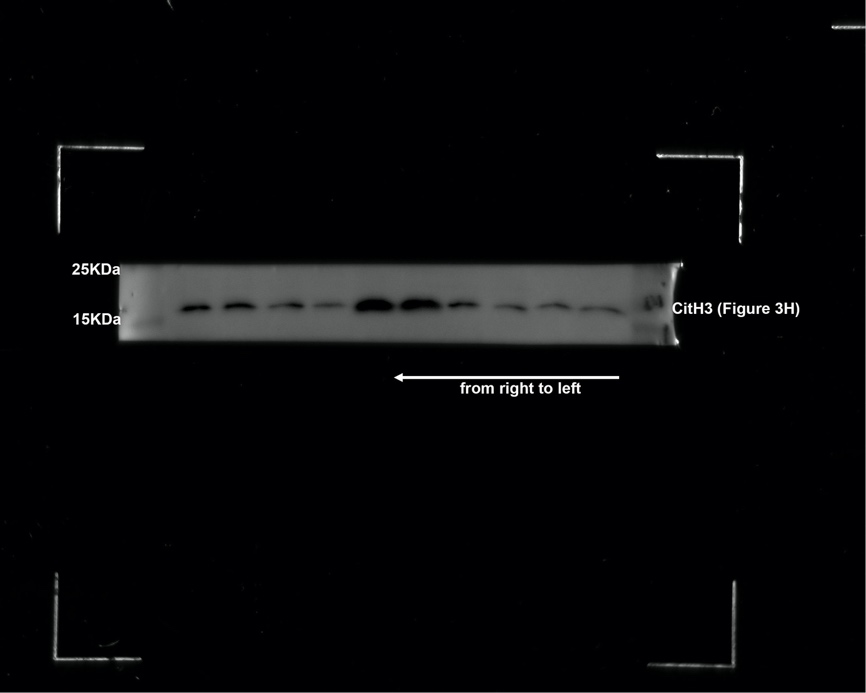


**c**


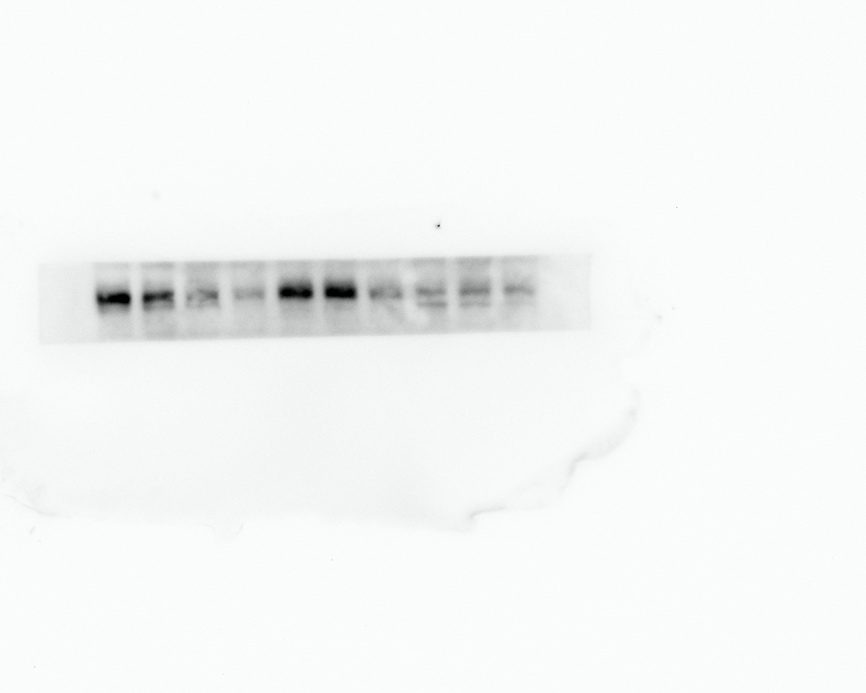


**d**


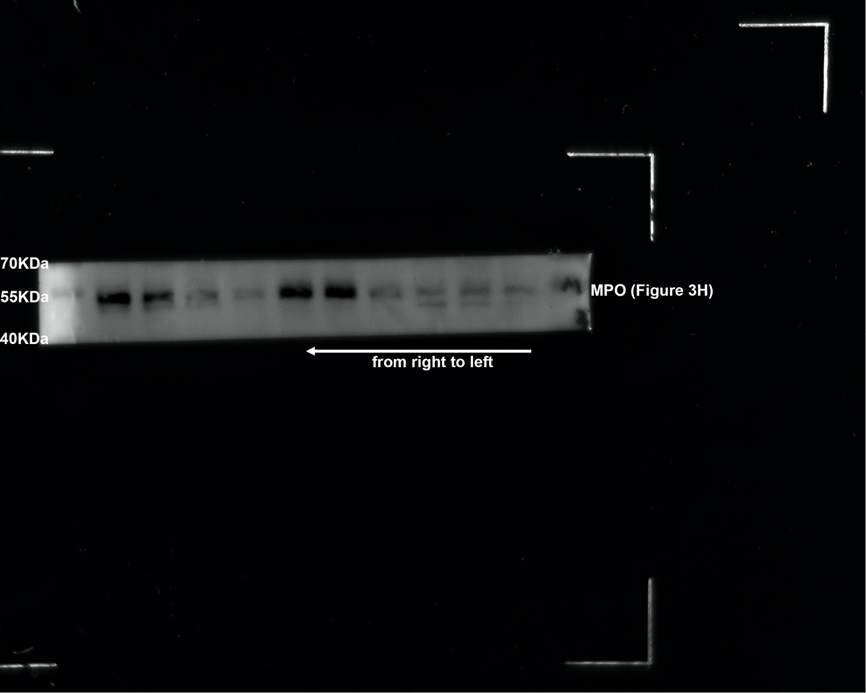


**e**


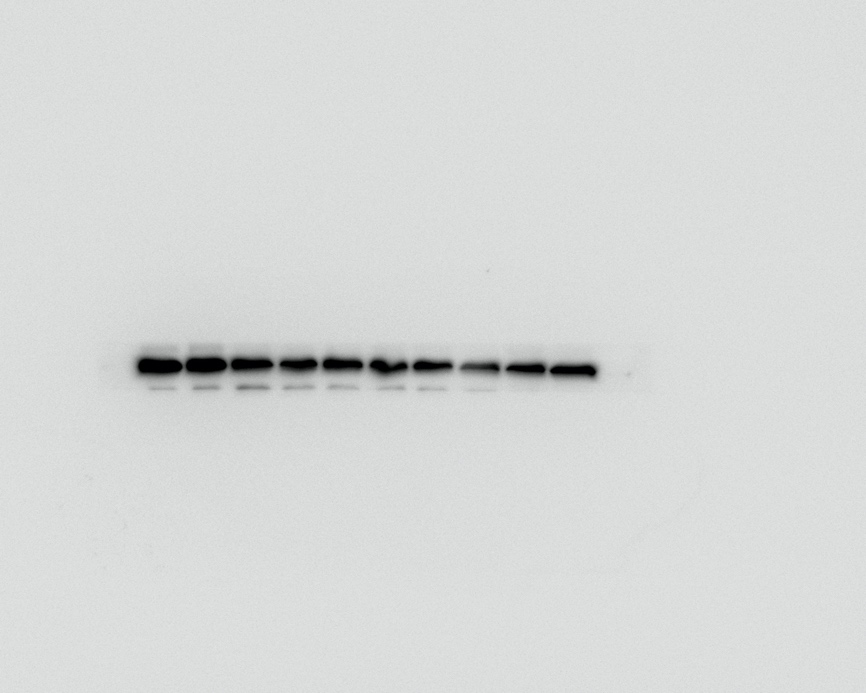


**f**


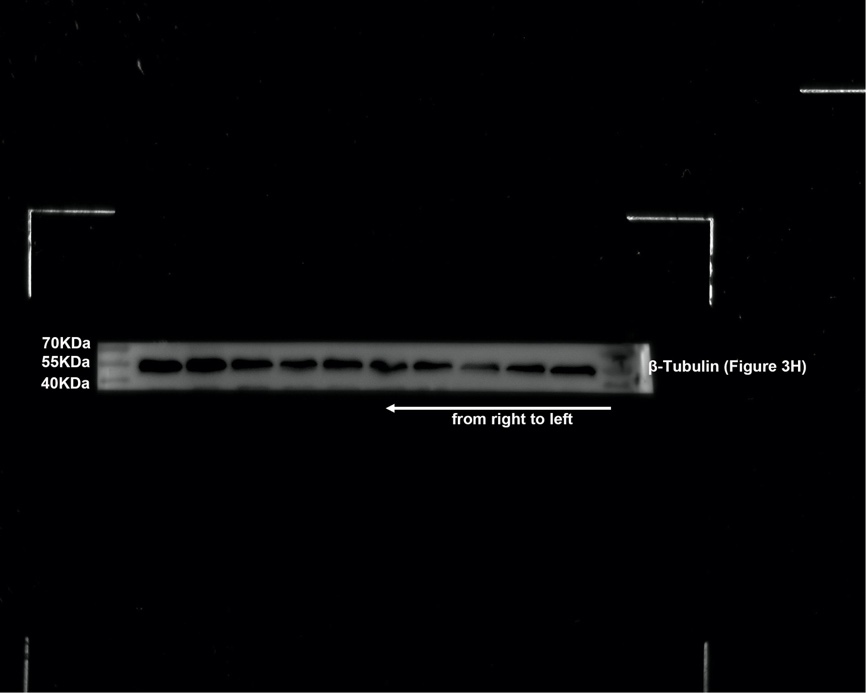


Supplementary Figure 4. Additional bands used for statistics in Figure 3H. CitH3 and β-Tubulin were separated by 15% SDS-PAGE, and MPO was separated by 12% SDS-PAGE. (a) (b) for CitH3; (c) (d) for MPO; (e) (f) for β-Tubulin.

a

**
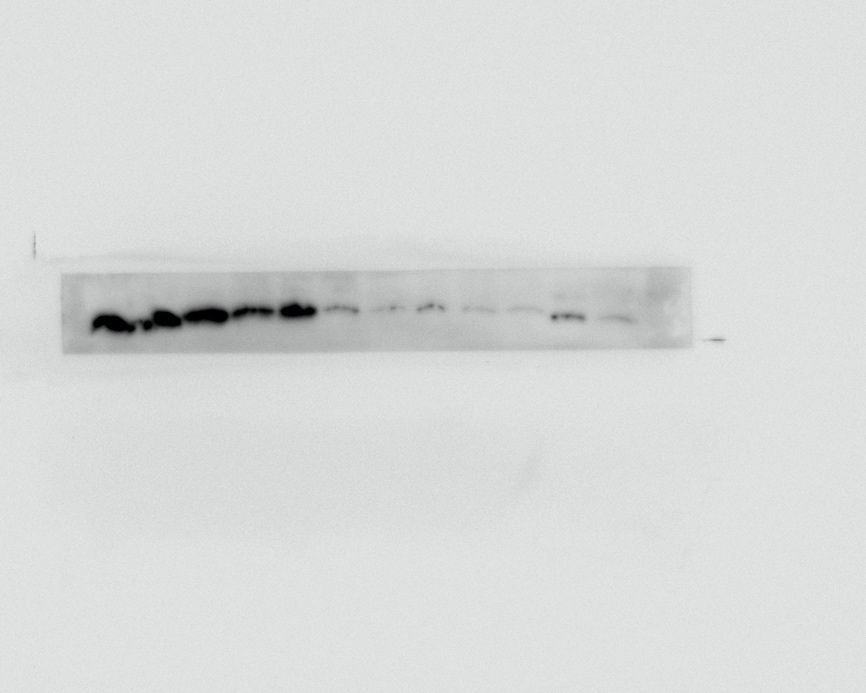
**

**b**

**
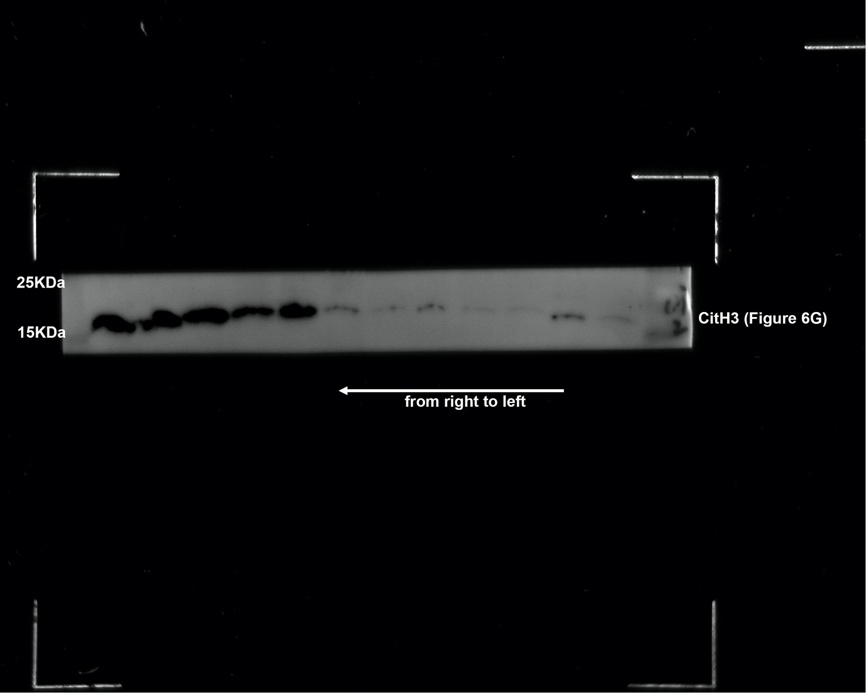
**

**c**

**
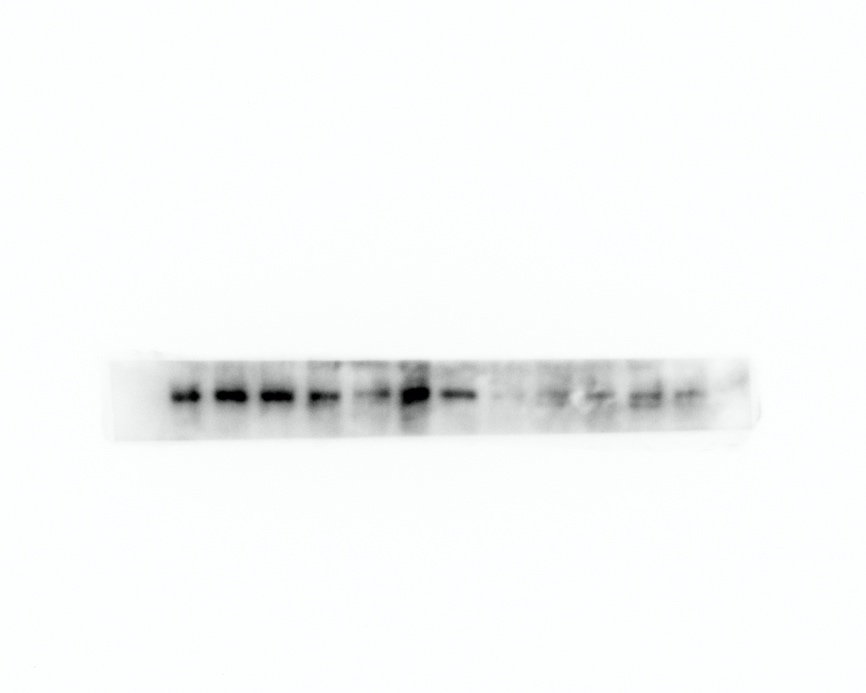
**

**d**

**
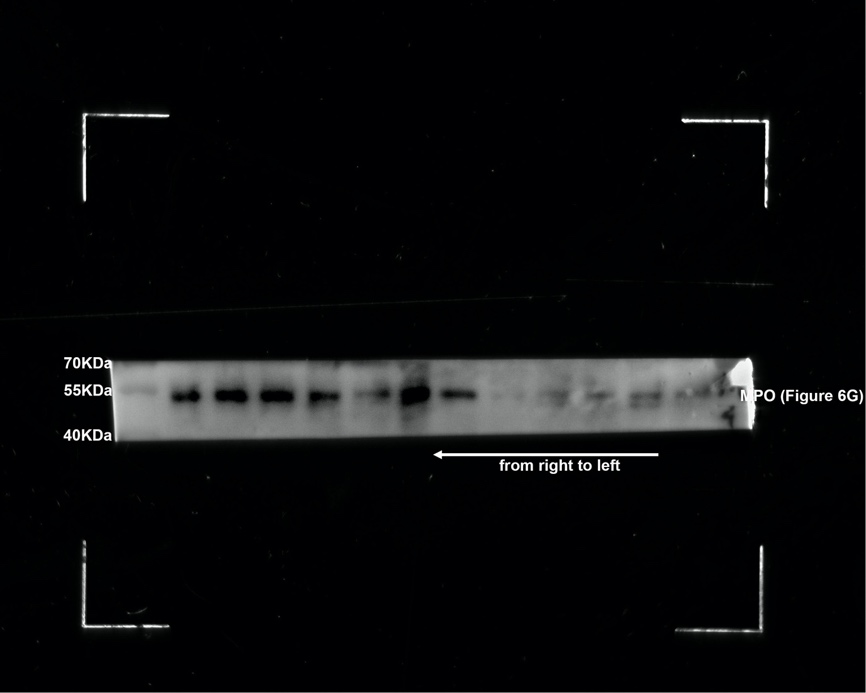
**

**e**

**
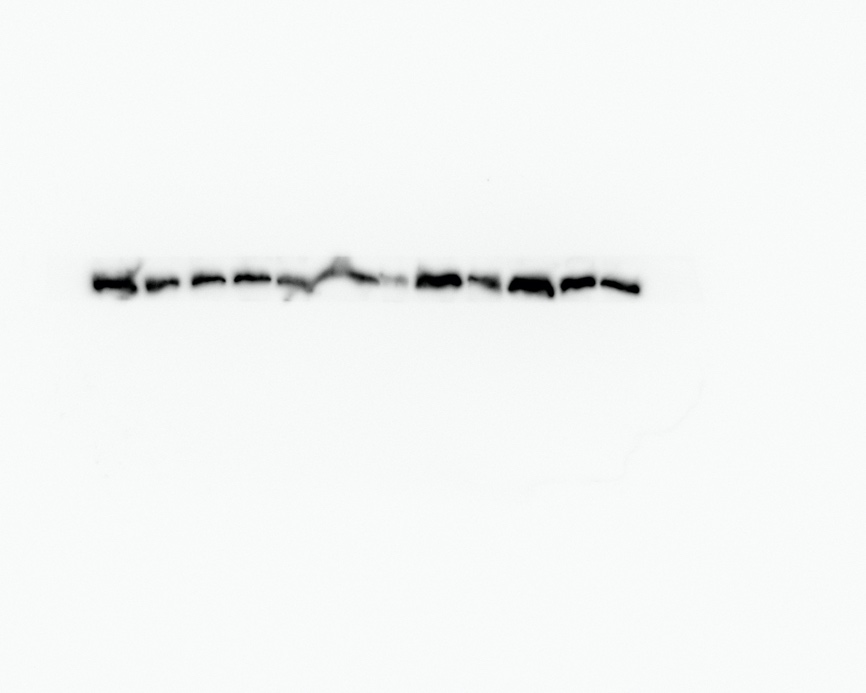
**

**f**

**
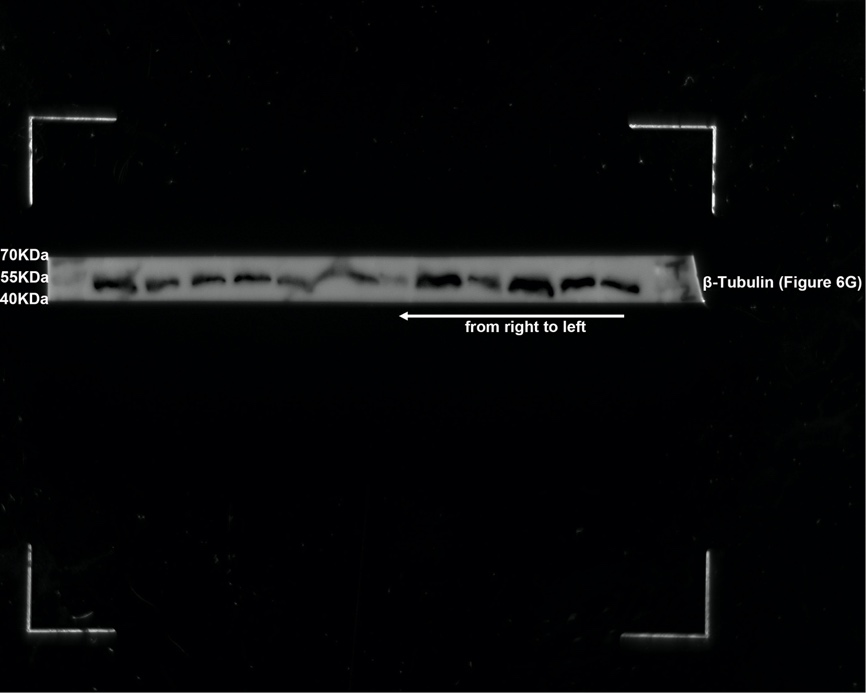
**

Supplementary Figure 5. Additional bands used for statistics in Figure 6G. CitH3 and β-Tubulin were separated by 15% SDS-PAGE, and MPO was separated by 12% SDS-PAGE. (a) (b) for CitH3; (c) (d) for MPO; (e) (f) for β-Tubulin.

**a**

**
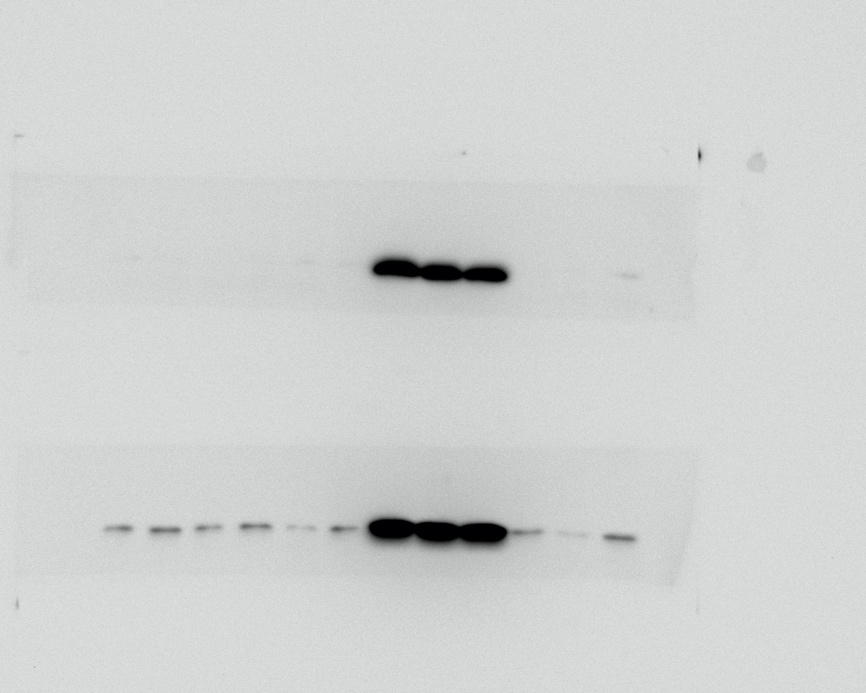
**

**b**

**
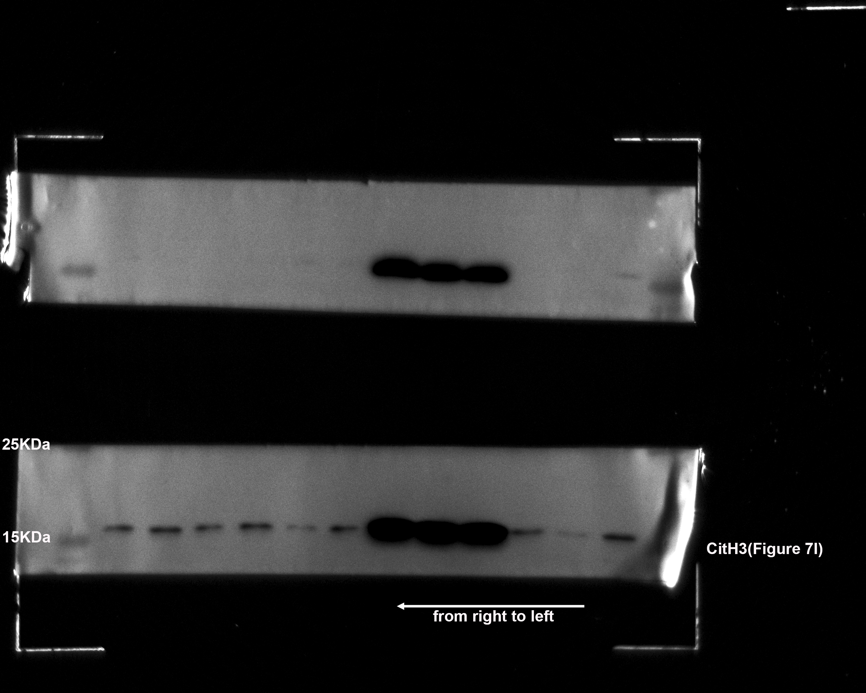
**

**c**

**
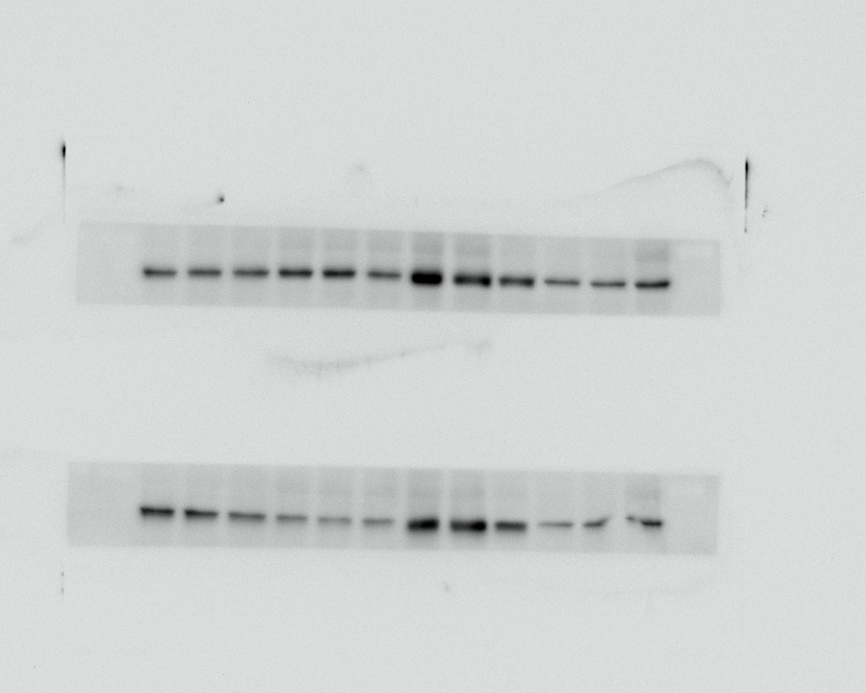
**

**d**

**
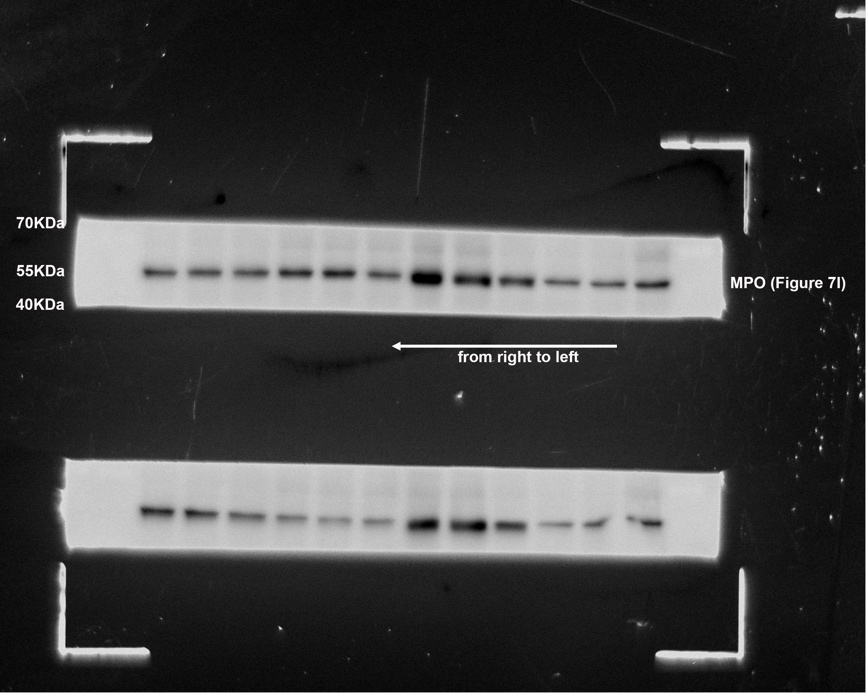
**

**e**

**
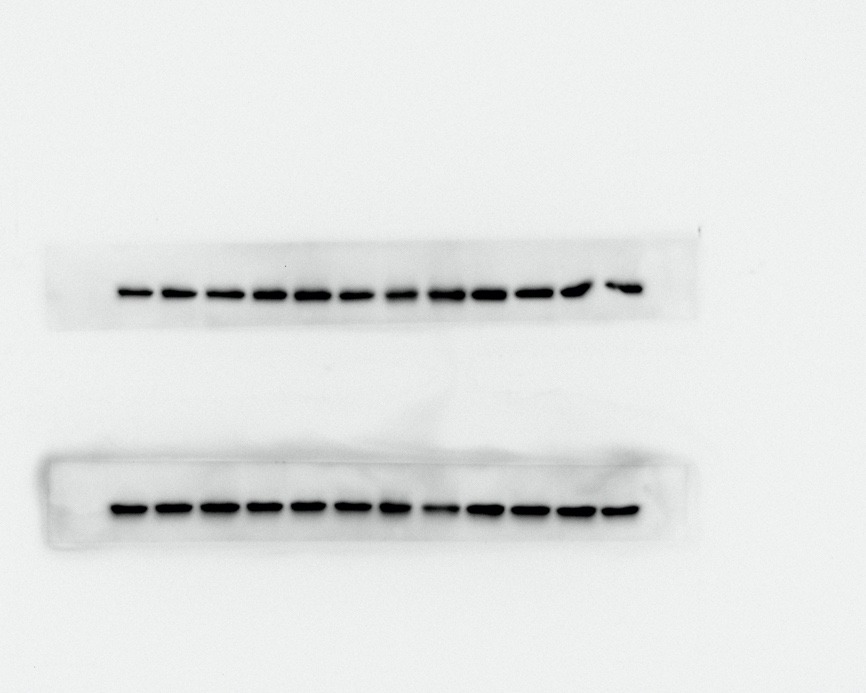
**

**f**

**
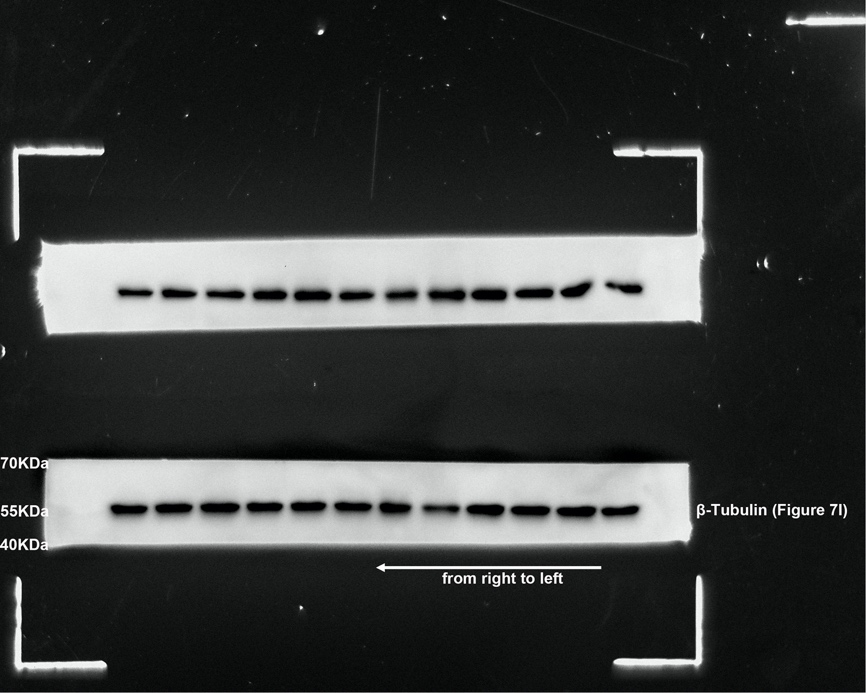
**

Supplementary Figure 6. Additional bands used for statistics in Figure 7I. Proteins were separated by 12% SDS-PAGE. (a) (b) for CitH3; (c) (d) for MPO; (e) (f) for β-Tubulin.


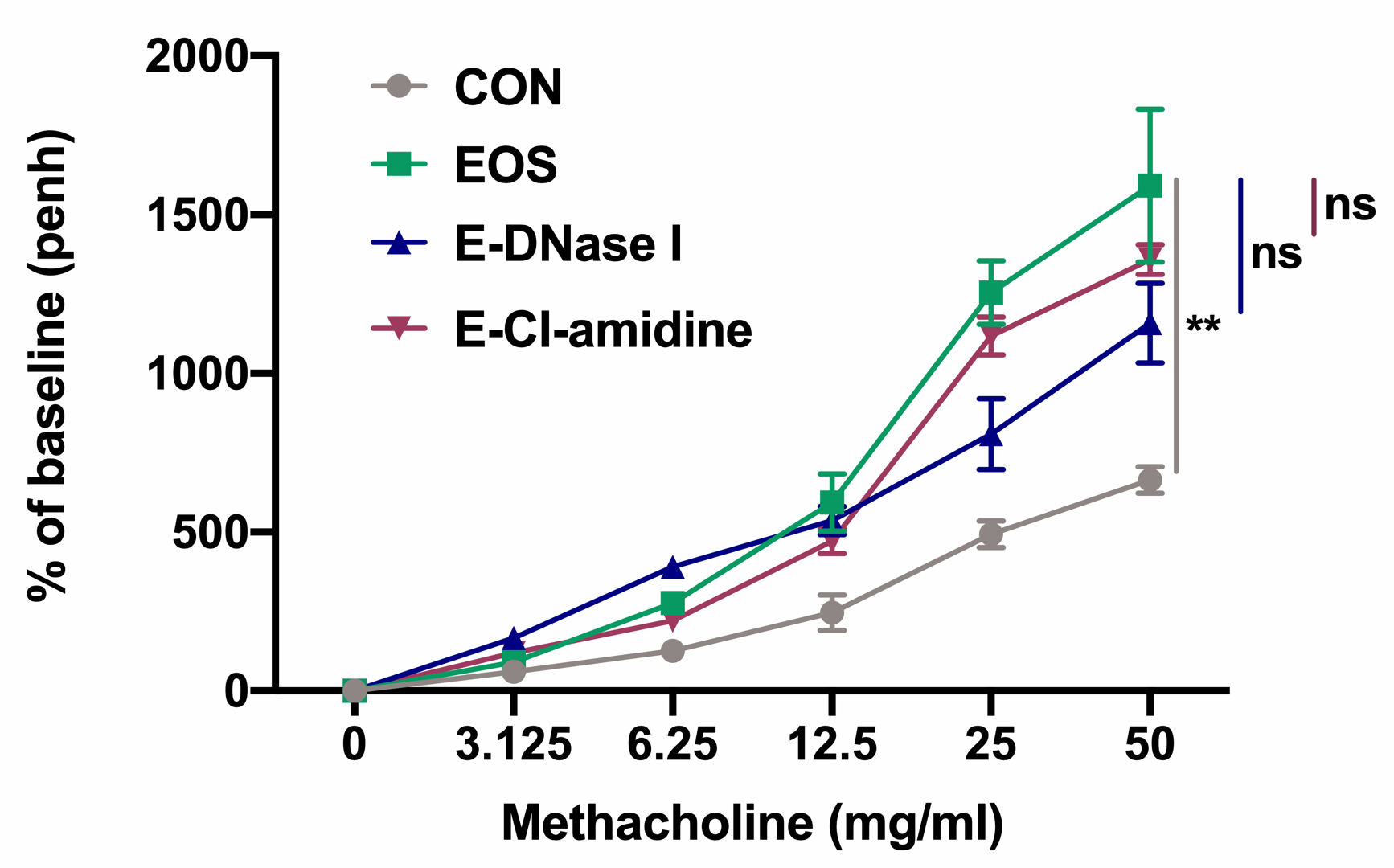
Supplementary Figure 7. The effects of DNase I and CI-amidine on AHR of the EOS group.
